# Solvent- and Co-Catalyst-Free Cycloaddition of Carbon Dioxide and Epoxides Catalyzed by Recyclable Bifunctional Niobium Complexes

**DOI:** 10.3390/ma16093531

**Published:** 2023-05-04

**Authors:** Qin Wen, Xuexin Yuan, Qiqi Zhou, Hai-Jian Yang, Qingqing Jiang, Juncheng Hu, Cun-Yue Guo

**Affiliations:** 1 Key Laboratory of Catalysis and Energy Materials Chemistry of Ministry of Education, Hubei Key Laboratory of Catalysis and Materials Science, College of Chemistry and Materials Science, South-Central Minzu University, Wuhan 430074, China; 2 School of Chemical Sciences, University of Chinese Academy of Sciences, Beijing 100049, China

**Keywords:** bifunctional niobium complex, carbon dioxide, epoxides, cyclic carbonate, recyclability

## Abstract

CO_2_, as a cheap and abundant renewable C1 resource, can be used to synthesize high value-added chemicals. In this paper, a series of bifunctional metallic niobium complexes were synthesized and their structures were characterized by IR, NMR and elemental analysis. All of these complexes have been proved to be efficient catalysts for the coupling reaction of CO_2_ and epoxides to obtain cyclic carbonates under solvent- and co-catalyst-free conditions. By using CO_2_ and propylene oxide as a model reaction, the optimal reaction conditions were systematically screened as: 100 °C, 1 MPa, 2 h, ratio of catalyst to alkylene oxide 1:100. Under the optimal reaction conditions, the bifunctional niobium catalysts can efficiently catalyze the coupling reaction with high yield and excellent selectivity (maximum yield of >99% at high pressure and 96.8% at atmospheric pressure). Moreover, this series of catalysts can also catalyze the coupling reaction at atmospheric pressure and most of them showed high conversion of epoxide. The catalysts have good substrate suitability and are also applicable to a variety of epoxides including diepoxides and good catalytic performances were achieved for producing the corresponding cyclic carbonates in most cases. Furthermore, the catalysts can be easily recovered by simple filtration and reused for at least five times without obvious loss of catalytic activity and selectivity. Kinetic studies were carried out preliminarily for the bifunctional niobium complexes with different halogen ions (3a(Cl^−^), 3b(Br^−^), 3c(I^−^)) and the formation activation energies (*E*_a_) of cyclic carbonates were obtained. The order of apparent activation energy *E*_a_ is 3a (96.2 kJ/mol) > 3b (68.2 kJ/mol) > 3c (37.4 kJ/mol). Finally, a possible reaction mechanism is proposed.

## 1. Introduction

In the last one to two centuries, with the large-scale application of fossil energy sources such as coal, oil and natural gas in countries around the world, a large amount of greenhouse gases has been released and the CO_2_ content in the atmosphere has increased, which has led to an increase in global temperature and frequent global extreme weather [1]. Meanwhile, CO_2_, as an abundant non-toxic, cheap and easily available C1 resource, can be used to synthesize a series of industrial products with high added value [2], which provides an idea to alleviate environmental problems. CO_2_ can be used as a building block to construct C–C, C–O, and C–N bonds for the synthesis of methanol [3], cyclic carbonates [4], oxazolidinones [5], and amides [6], which are important chemical intermediates and pharmaceutical intermediates. Among them, the construction of C–O bonds is a focus as well as a hot spot of current research.

Through the reaction of CO_2_ with epoxy compounds, cyclic carbonate can be obtained, which is a class of organic solvents with excellent performance and important fine chemical intermediates, and its application is very wide and the demand is particularly high [7,8,9]. In response to the market demand, a variety of catalytic systems have been developed, such as Al, Mg, Zn, and other metal catalysts [10,11,12,13], ionic liquids [14], azacyclic carbines [15], 1,5,7-triazabicyclo[4.4.0]dec-5-ene (TBD) [16] and other catalytic systems, all of which can catalyze the coupling reaction of CO_2_ with epoxides with excellent catalytic performance. Among them, metal complexes are one of the most utilized catalysts, mainly due to three advantages: (1) the wide scope of metal centers, which allows the preparation of a large number of metal complexes, (2) facile preparation from commercially available and relatively inexpensive starting materials, and (3) relatively high catalytic efficiency [11,12,13].

So far, many metal complexes (e.g., Zn, Ni, Rh, Ir, Fe, Cu, Re, Al, Co, Cr, Pb, Mg, Nb, etc.) are mainly used as catalysts for the coupling reaction of epoxides with CO_2_ [17,18,19,20]. It has been known that niobium-containing materials are presently of great interest in heterogeneous catalysis where they are used as catalyst components. As a nontoxic, inexpensive metal, niobium complexes have also been used to catalyze the conversion of CO_2_ and epoxides into carbonates. Although Kühn and co-workers have proved several niobium-based catalysts to be efficient in the catalytic conversion of carbon dioxide and epoxides into carbonates under mild conditions, the addition of suitable nucleophiles as co-catalysts is required in order to maintain high activity [21,22,23]. Until 2015, Hou’s group reported a series of peroxoniobate salts of organic bases as a halogen-free, air-stable, recyclable and single-component catalyst for the cycloaddition reaction of epoxides and CO_2_ [24]. The work represented a simple, ecologically friendly and efficient route for CO_2_ chemical fixation into high value chemicals and exhibited the unique advantages of niobium-based catalysts. However, there has been almost no work reported about niobium-based catalysts, especially with bifunctionalization ability, for the coupling reaction of CO_2_ and epoxides. 

Herein, a series of bifunctional niobium complexes were designed and synthesized to catalyze the synthesis of cyclic carbonates from CO_2_ and epoxides under solvent-free and co-catalyst-free conditions. The catalytic activities of different complexes and the optimization of the reaction conditions were also investigated.

## 2. Materials and Methods

### 2.1. Chemicals and Analytical Methods

All of the chemicals were purchased from Acros.com and used as received except for the epoxides, which were purified by distillation from CaH_2_ before utilization. Copies of all spectra of synthesized complexes and carbonate products are provided in the Supplementary Materials (SM).

A Bruker Al-400 MHz instrument manufactured by Bruker Technologies Switzerland Ltd., Fällanden, Switzerland, was used for recording NMR spectra using TMS as an internal standard. A Perkin-Elmer 2000 FT-IR spectrometer (manufactured by Perkin-Elmer Ltd., Waltham, MA, USA) was used for IR spectra record. Elemental analysis was conducted on a PE 2400 series II CHNS/O elemental analyzer (manufactured by Perkin-Elmer Ltd., Waltham, MA, USA).

### 2.2. Synthesis of Bifunctional Niobium Complexes

**Scheme 1:**
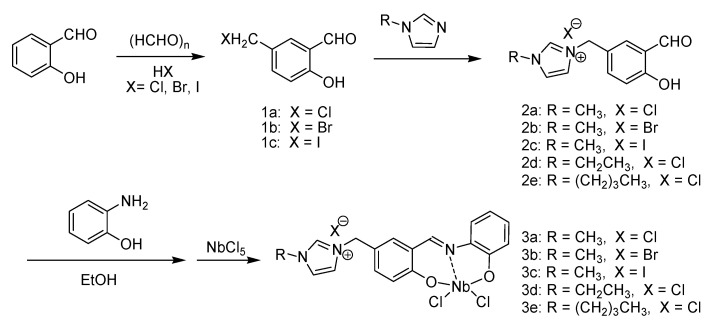
Synthesis of bifunctional niobium complexes.

The synthetic route of bifunctional niobium complexes is shown in Scheme 1. The compounds 1a–1c and 2a–2e are known and were synthesized according to the literature [25,26,27].

Synthesis of compound 3: 5.0 g of compound 2, 2.16 g of *o*-aminophenol and 5.36 g of NbCl_5_ were dissolved in 20 mL of ethanol in the flask. After stirring for 24 h at room temperature, the produced solid was filtered and washed with ethanol and acetone several times and dried under vacuum to obtain the corresponding bifunctional niobium complex.

3a: pale yellow powder, yield 82%, melting point > 300 °C. ^1^H NMR (400 MHz, DMSO) δ 10.28 (s, 1H), 7.73 (dd, J = 23.0, 12.6 Hz, 3H), 7.59 (d, J = 8.8 Hz, 1H), 7.46 (s, 1H), 7.32 (d, J = 8.2 Hz, 1H), 7.20 (d, J = 8.7 Hz, 1H), 7.09 (d, J = 8.5 Hz, 1H), 7.07–6.95 (m, 1H), 6.87 (d, J = 7.1 Hz, 1H), 5.35 (d, J = 12.0 Hz, 2H), 3.83 (d, J = 6.3 Hz, 3H); ^13^C NMR (101 MHz, DMSO) δ 190.69, 161.71, 151.32, 136.95, 129.55, 124.71, 124.41, 122.59, 119.51, 40.61, 40.40, 40.20, 39.99, 39.93, 39.78, 39.57, 39.36, 36.32; Selected IR peaks (KBr, cm^−1^): ν 3383, 3092, 2920, 2686, 2568, 1604, 1475, 1358, 1301, 1254, 1168, 1025, 881, 770, 621. Anal. Calcd for C_18_H_16_Cl_3_N_3_NbO_2_: C, 42.76; H, 3.19; N, 8.31. Found: C, 42.96; H, 3.35; N. 8.21.

3b: green powder, yield 71%, melting point >300 °C. ^1^H NMR (400 MHz, DMSO) δ 10.23 (d, J = 38.8 Hz, 1H), 7.86–7.68 (m, 3H), 7.62 (d, J = 8.6, 2.2 Hz, 1H), 7.51 (d, J = 8.5, 2.1 Hz, 1H), 7.39 (d, J = 7.8 Hz, 1H), 7.25–7.14 (m, 1H), 7.10 (d, J = 16.0, 6.8 Hz, 1H), 7.00 (d, J = 8.5 Hz, 1H), 6.85 (dt, J = 15.1, 7.5 Hz, 1H), 5.38 (d, J = 15.1 Hz, 2H), 3.84 (d, J = 7.6 Hz, 3H); ^13^C NMR (101 MHz, DMSO) δ 186.55, 161.82, 140.05, 137.00, 132.25, 124.42, 123.54, 122.60, 122.06, 119.59, 119.30, 117.96, 105.27, 51.79, 40.63, 40.57, 40.42, 40.36, 40.22, 40.15, 40.01, 39.94, 39.80, 39.74, 39.59, 39.53, 39.38, 39.32, 36.45, 36.32; Selected IR peaks (KBr, cm^−1^): ν 3383, 3093, 2921, 2686, 2568, 1642, 1474, 1358, 1299, 1253, 1167, 1024, 881, 768, 620. Anal. Calcd for C_18_H_16_BrCl_2_N_3_NbO_2_: C, 39.30; H, 2.93; N, 7.64. Found: C, 39.02; H, 2.85; N. 7.53.

3c: brown powder, yield 67%, melting point >300 °C. ^1^H NMR (400 MHz, DMSO) δ 10.28 (s, 1H), 7.77 (dd, J = 38.3, 9.1 Hz, 3H), 7.62 (d, J = 8.6 Hz, 1H), 7.49 (d, J = 6.9 Hz, 1H), 7.37 (t, J = 7.8 Hz, 1H), 7.18 (d, J = 8.3 Hz, 1H), 7.09 (d, J = 16.5, 8.6 Hz, 1H), 7.05–6.93 (m, 1H), 6.85 (d, J = 9.5 Hz, 1H), 5.38 (d, J = 14.1 Hz, 2H), 3.84 (d, J = 7.0 Hz, 3H); ^13^C NMR (101 MHz, DMSO) δ 191.03, 161.63, 151.11, 137.06, 136.91, 129.45, 126.08, 124.41, 122.92, 122.65, 122.55, 120.13, 119.78, 118.59, 118.01, 116.64, 51.56, 40.49, 40.28, 40.07, 39.87, 39.66, 39.45, 39.24, 36.40, 36.34; Selected IR peaks (KBr, cm^−1^): ν 3417, 3092, 1637, 1476, 1384, 1289, 1253, 1163, 1018, 751, 620. Anal. Calcd for C_18_H_16_ICl_2_N_3_NbO_2_: C, 36.21; H, 2.70; N, 7.04. Found: C, 36.10; H, 2.89; N. 7.34.

3d: yellow powder, yield 59%, melting point >300 °C. ^1^H NMR (400 MHz, DMSO) δ 10.24 (d, J = 31.4 Hz, 1H), 7.96–7.80 (m, 3H), 7.74 (d, J = 2.2 Hz, 1H), 7.67–7.54 (m, 1H), 7.51–7.37 (m, 1H), 7.21 (d, J = 8.5 Hz, 1H), 7.14 (d, J = 14.7, 4.9 Hz, 1H), 7.06 (d, J = 8.6 Hz, 1H), 6.90–6.76 (m, 1H), 5.39 (d, J = 19.2 Hz, 2H), 4.19 (p, J = 7.4 Hz, 2H), 1.40 (q, J = 7.7 Hz, 3H); ^13^C NMR (101 MHz, DMSO) δ 190.70, 161.86, 161.20, 151.51, 136.97, 136.22, 133.43, 129.55, 129.12, 126.01, 124.72, 122.97, 122.93, 122.72, 120.49, 120.02, 119.52, 119.40, 118.71, 118.04, 117.25, 116.81, 51.71, 51.58, 44.78, 40.61, 40.40, 40.32, 40.19, 40.11, 39.98, 39.90, 39.77, 39.70, 39.56, 39.49, 39.35, 15.44. Selected IR peaks (KBr, cm^−1^): ν 3380, 3136, 2987, 2692, 2573, 1643, 1509, 1473, 1358, 1299, 1252, 1167, 1022, 876, 771. Anal. Calcd for C_19_H_18_Cl_3_N_3_NbO_2_: C, 43.92; H, 3.49; N, 8.09. Found: C, 43.81; H, 3.34; N. 7.95.

3e: yellow powder, yield 51%, melting point >300 °C. ^1^H NMR (400 MHz, DMSO) δ 10.21 (d, J = 29.4 Hz, 1H), 7.84–7.76 (m, 3H), 7.68 (d, J = 1.8 Hz, 1H), 7.57–7.43 (m, 1H), 7.38–7.28 (m, 1H), 7.15 (d, J = 7.9 Hz, 1H), 7.07 (d, J = 15.8, 5.3 Hz, 1H), 6.98 (d, J = 8.9 Hz, 1H), 6.87–6.73 (m, 1H), 5.17 (d, J = 18.9 Hz, 2H), 4.07 (p, J = 6.5 Hz, 2H), 1.23 (q, J = 6.8 Hz, 2H), 0.48 (t, J = 5.9 Hz, 3H); ^13^C NMR (101 MHz, DMSO) δ 183.01, 151.18, 147.81, 129.10, 127.38, 124.30, 123.81, 122.89, 122.25, 120.12, 120.00, 119.62, 119.06, 116.72, 113.00, 49.21, 45.43, 40.63, 40.42, 40.21, 40.00, 39.79, 39.58, 39.38, 38.17, 31.75, 31.60, 19.30, 17.84, 13.74, 12.02. Selected IR peaks (KBr, cm^−1^): ν 3385, 2960, 2590, 1618, 1558, 1487, 1462, 1384, 1293, 1249, 1158, 760, 617. Anal. Calcd for C_21_H_22_Cl_3_N_3_NbO_2_: C, 46.05; H, 4.05; N, 7.67. Found: C, 46.21; H, 4.12; N. 7.78.

NMR spectra of the synthesized substances are included in Supplementary Materials (Figure S1).

### 2.3. Experimental Procedure for the Cycloaddition of Carbon Dioxide and Epoxides 

Reaction under high pressure: The quantitative epoxy compound and bifunctional niobium complex were added into a stainless steel reactor with a magnetic stir, then the reactor was sealed. CO_2_ was pressurized into the reactor to replace the gas three times before it was immersed into the oil bath with pre-set temperature for 20 min. The reaction started under stable CO_2_ pressure in the reactor. When the preset time was reached, the reaction vessel was cooled quickly with ice water to release the pressure slowly. After exhausting the gas, a small amount of the mixture was taken for ^1^H NMR characterization to calculate the yield and selectivity.

Reaction under atmospheric pressure: Under the protection of CO_2_, a quantitative amount of bifunctional niobium complex and stir bar were added into the Schlenk bottle connecting with a CO_2_ balloon sealed with rubber cap. Then, a quantitative amount of epoxide compound was injected into the bottle with a syringe. The reaction system was preheated in a constant temperature oil bath for 20 min and then the reaction was started. When the required reaction time was reached, the reactor was cooled quickly with ice water to release the pressure slowly, and then a small amount of the mixture was taken with a syringe for ^1^H NMR analysis and the yield and selectivity were calculated.

## 3. Results and Discussion

### 3.1. Optimization of Reaction Conditions

**Figure 1:**
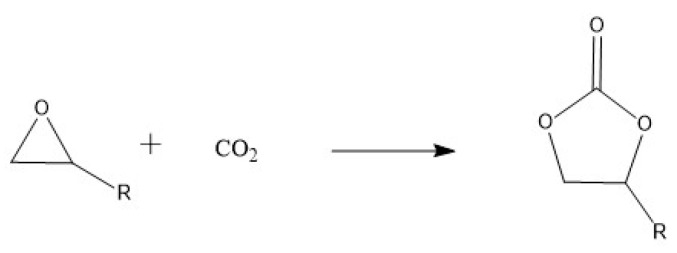
Synthesis of cyclic carbonate.

The synthetic route of cyclic carbonate is shown in Figure 1. The effect of catalyst type, catalyst dosage, temperature, time and CO_2_ pressure on the reaction was investigated systematically, and ultimately the optimum reaction conditions were explored, under which a variety of epoxides were catalyzed to investigate the catalytic efficiency and substrate suitability of the catalytic system.

#### 3.1.1. *Effect of Catalyst Type on Catalytic Activity*

Firstly, the effect of anion halogen ions on catalyst activity was tested under the conditions of 100 °C, 1 MPa, 2 h, catalyst:epoxide = 1:100 (Table 1). It can be seen that there is no propylene carbonate (PC) produced without the catalyst. Ionic liquids have been proved as efficient catalysts, so the catalytic activities of ligands 2a–2e have also been performed and good results were obtained (Entries 2–6). However, the recyclability of these ligands is worse compared with their metal complexes. The catalytic activities of three niobium complexes 3a–3c were proved as efficient catalysts showing almost the same activity within 2 h. To clarify the catalytic performance of 3a–3c, shorter time reactions of 1 h have been investigated and there are still no big differences for their catalytic activities as shown in Table 1 (Entries 13–15). Due to the lower yields for the preparation of catalysts with Br^−^ and I^−^ ions, the catalyst **3a** containing Cl^-^ ions were chosen for the following studies. The effect of the alkyl substituent on the imidazole moiety of catalyst activity was then investigated (Entries 7,10,11). The length of the alkyl chain showed a relatively significant effect on the catalyst activity. The best yields were obtained when the substituent was methyl (3a). The reason for this occurrence may be due to the effect of spatial hindrance, the greater the spatial hindrance, the lower the catalytic activity. So 3a was chosen as the optimal catalyst for the coupling reaction of CO_2_ and epoxide.

#### 3.1.2. *Effect of Reaction Parameters on Catalytic Activity*

**Figure 2:**
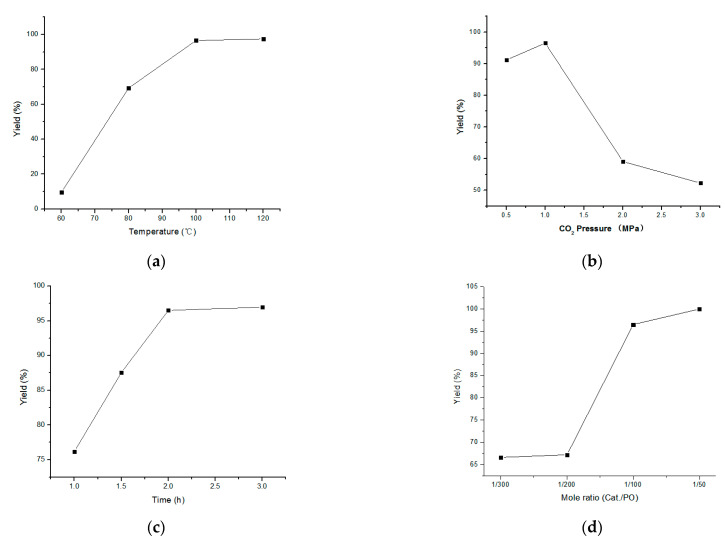
Effect of reaction parameters on reaction activity. (**a**) Effect of temperature: propylene oxide (5 mL, 0.0714 mol), catalyst (0.714 mmol), 1 MPa, 2 h. (**b**) Effect of CO_2_ pressure: propylene oxide (5 mL, 0.0714 mol), catalyst (0.714 mmol), 100 °C, time 2 h. (**c**) Effect of time: propylene oxide (5 mL, 0.0714 mol), catalyst (0.714 mmol), 100 °C, 1 MPa. (**d**) Effect of catalyst dosage: propylene oxide (5 mL, 0.0714 mol), 100 °C, 1 MPa, 2 h. The selectivity of cyclic carbonate for all reactions was above 99%.

After determining the type of catalyst, the coupling reaction of propylene oxide and carbon dioxide was used as a model reaction to explore the effects of temperature, CO_2_ pressure, time, and catalyst dosage on the reaction activity (Figure 2).

As shown in Figure 2a, low temperature resulted in lower activity. When the temperature gradually increases, the product yield continues to increase. Although the yield is a little better at 120 °C than that obtained at 100 °C, the increase is not obvious. The cycloaddition reaction of CO_2_ with epoxide is exothermic, so from the viewpoint of thermodynamic equilibrium, too high temperature will hinder the formation of cyclic carbonate [28,29]. In addition, high temperature also leads to the polymerization of cyclic carbonate, which reduces the catalytic efficiency [30]. Therefore, 100 °C is selected as the optimal reaction temperature.

The effect of CO_2_ pressure on the catalytic activity is shown in Figure 2b. The product yield showed a trend of first rising and then decreasing with the CO_2_ pressure and the highest activity was obtained at 1 MPa. The pressure is increased first because of the increase in CO_2_ concentration involved in the reaction. Therefore, the yield showed an upward trend at the initial stage. When the pressure increases to a certain value, too high CO_2_ pressure would decrease the propylene oxide (PO) concentration in the vicinity of the catalyst to lower PC yield. These opposite factors’ competition gave rise to an optimal pressure of 1 MPa for the best PC yields [31,32].

Time is another indispensable factor and its effect is shown in Figure 2c. The yield of cyclic carbonate increases with time. At the early stage, the yield increases sharply and almost linearly, but the yield hardly increases when the time exceeds 2 h, so it is appropriate to choose the optimal reaction time as 2 h.

In addition to these factors, the influence of the amount of catalyst on the reaction activity is also important. As shown in Figure 2d, when the amount of catalyst is at a lower level, the product yield is relatively low too. When the ratio of the amount of substrate to catalyst reaches 1:100, the product yield reaches the maximum 96%. However, the increase in product yield is not obvious when the amount of catalyst continues to increase. So, the ratio of catalyst to alkylene oxide 1:100 is selected as the optimal value.

In summary, the optimal conditions for the bifunctional niobium complex for the cycloaddition of CO_2_ and epoxide were screened as: reaction temperature of 100 °C, carbon dioxide pressure of 1 MPa, reaction time of 2 h and catalyst to epoxide ratio of 1:100.

### 3.2. Applicability of Substrates

To investigate the suitability of the catalytic system for more substrates expansion, various epoxides were tested for the coupling reaction both at high and atmospheric pressure and the results are shown in Table 2.

Under optimal reaction conditions (100 °C, 1 MPa, 2 h, 1:100 catalyst to epoxide ratio), the bifunctional niobium complex can efficiently catalyze the cycloaddition reactions of a wide range of epoxides with CO_2_. The yields of cyclic carbonate for epoxides with relatively low spatial hindrance, such as epichlorohydrin, 2-(isopropoxymethyl)oxirane, 2-phenyloxirane and 2-butyloxirane, the corresponding yields of cyclic carbonates are 100%, 96.4%, 90.9%, and 74.8%, respectively (entries 1–4), but for epoxides with bigger steric hindrance, such as cyclohexene oxide and 2,2-dimethyloxirane, the reaction was extended to 12 h with only moderate yields (Table 2, entries 5,6). It is worth noting that this catalyst is also suitable for bis-epoxides, which can be obtained in excellent yields (entries 7,8). The bicyclic carbonate synthesized from the bis-epoxides has an important role in industry, which is a feedstock for the reaction with polyfunctional primary amines to produce non-isocyanate polyurethanes (NIPUs). The present catalyst is also suitable for substrates of the glycidyl ether family, showing excellent catalytic activity (entries 9–12).

The catalytic performance of the bifunctional niobium complex for various epoxides was also tested at atmospheric pressure and 100 °C (Table 2, condition b; most boiling points of the substrates studied in our work are above 100 °C except for 2,2-dimethyloxirane (entry 6)). Epichlorohydrin can be converted almost completely within 14 h (entry 1), and for other epoxides with low steric hindrance, relatively good yields were obtained within 24 h (Table 2, entries 2–4). However, for epoxides with high steric hindrance, yields were still poor even after 24 h reaction (entry 5). For the diepoxy alkane compounds, on the other hand, moderate or even excellent yields could be achieved under reaction conditions (entries 7–8). For glycidyl ether compounds, except for phenyl glycidyl ether, excellent yields can be achieved within 11 h. All other glycidyl ethers required longer reaction times to achieve excellent yields (entries 9–12).

In summary, the catalytic system has good substrate suitability and can catalyze the reaction of a variety of epoxides with CO_2_ to form the corresponding cyclic carbonates under both high pressure and atmospheric conditions with satisfactory results.

### 3.3. Kinetic Study

**Figure 3:**
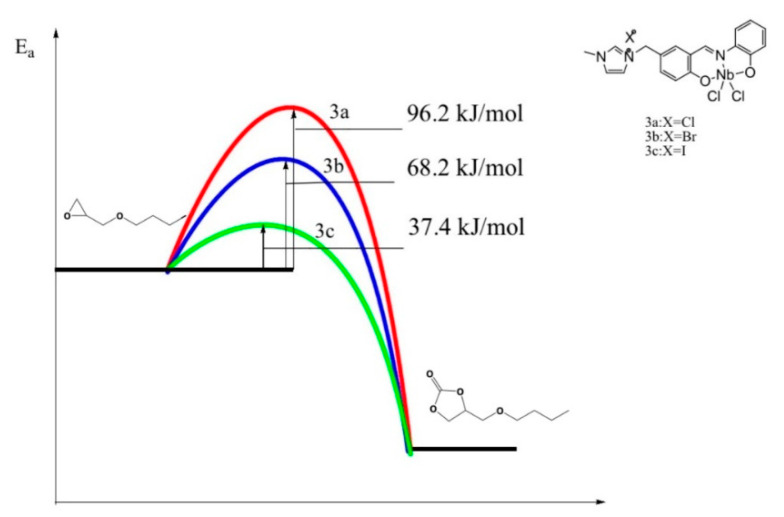
The apparent activation energy *E*_a_ of the reaction between carbon dioxide and *n*-butyl glycidyl ether catalyzed by the bifunctional niobium complexes with different halogen ions.

The kinetics of the carbon dioxide cycloaddition reaction catalyzed by bifunctional niobium complexes was also investigated in detail. The cycloaddition of *n*-butyl glycidyl ether (BGE) and CO_2_ was selected as model reaction at atmospheric pressure, and the kinetic behavior of bifunctional niobium complexes containing different halogen ions was studied in the temperature range of 353–413 K and the reaction time range of 2–8 h (please refer to the Supplementary Materials). The apparent activation energy *E*_a_ of the bifunctional niobium complexes with different halogen ions are shown in Figure 3. The order of apparent activation energy *E*_a_ is 3a (96.2 kJ/mol) > 3b (68.2 kJ/mol) >3c (37.4 kJ/mol) (Figure 3), which is consistent with the yields of 96%, 97% and 99% of cyclic carbonate formation from PC and CO_2_ catalyzed by 3a, 3b, and 3c, respectively. This is attributed to the leaving ability of the contained halogen ions (Cl^−^ < Br^−^ < I^−^).

### 3.4. Reusability of Catalyst

**Figure 4:**
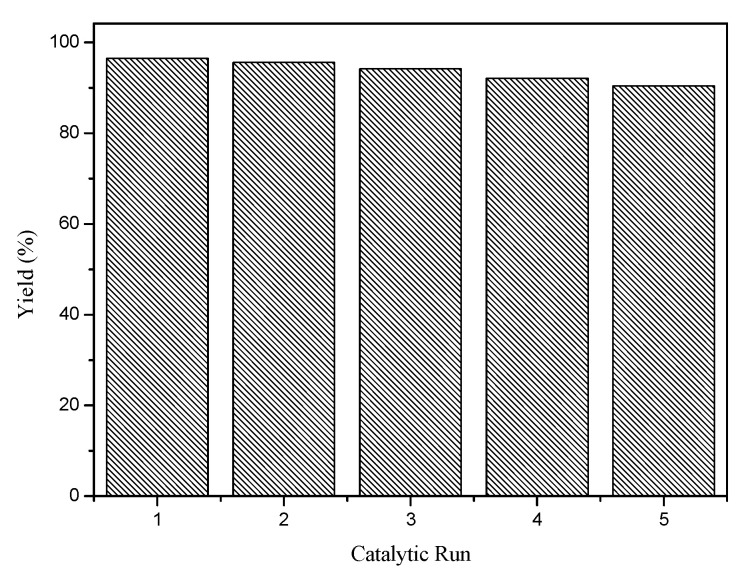
Recycling of bifunctional niobium complex 3a. (Conditions: PO 5 mL, 0.0714 mol; catalyst 0.714 mmol; 100 °C; 1 MPa; 2 h. All the selectivities are >99%).

**Figure 5:**
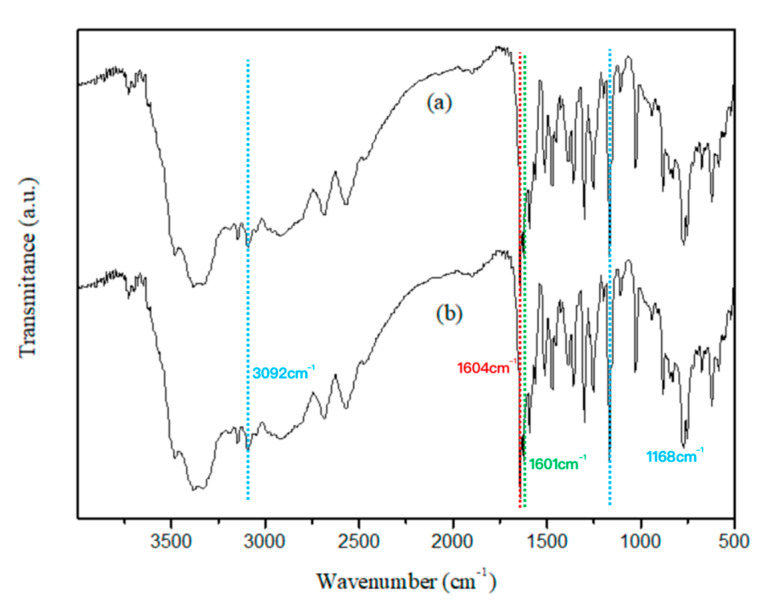
IR spectra of catalyst 3a before and after 5 repetitions (**a**) Fresh catalyst 3a (**b**) Catalyst 3a after five repetitions.

The recyclability of the catalyst was investigated using propylene oxide as a template reaction with CO_2_ under optimal reaction conditions. After each cycle of reaction, acetone was added to the reaction system to precipitate the catalyst out of the mixture. After filtration and drying under vacuum, the catalyst was reused. The catalyst can be reused at least for five times without obvious loss of catalytic activity and selectivity (Figure 4). The fresh catalyst and the catalyst after five reactions were selected for IR characterization and the results are shown in Figure 5. The IR spectra indicate that the catalyst was stable even after five cycles. As shown in Figure 5, the typical peaks for the imidazole functionalized complex **3a** are visualized clearly and all of them show no significant change before and after five reaction cycles: the peak of 1604 cm^−1^ is the absorption peak of C=N of Schiff base, the peak of 1601 cm^−1^ belongs to benzene ring skeleton, and the absorption peaks of 3092 cm^−1^and 1172 cm^−1^ are attributed to the C-H stretching vibration on the imidazole cation and the stretching vibration peak of the imidazole ring, respectively.

### 3.5. Possible Reaction Mechanisms

**Figure 6:**
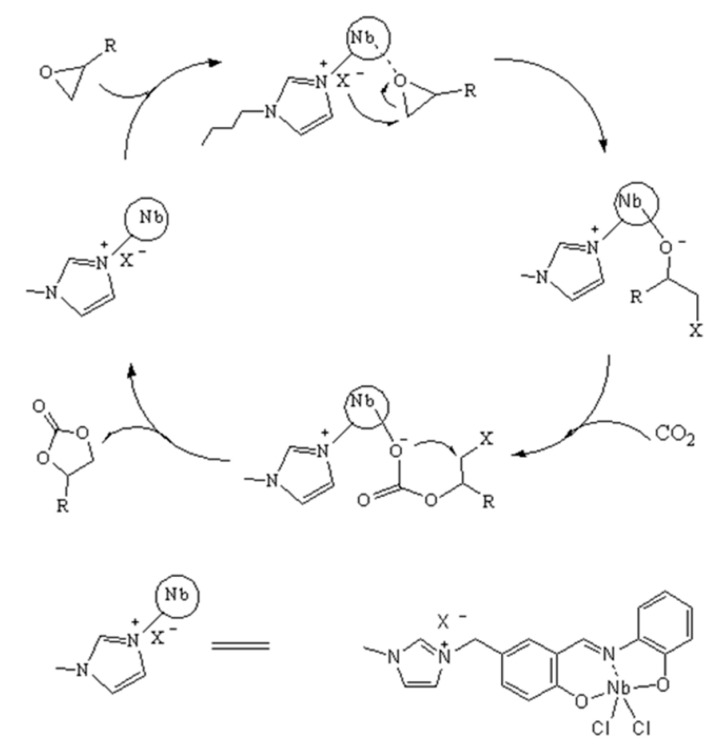
Possible reaction mechanism.

Based on previously reported literature and experimental results [33,34,35,36], a possible reaction mechanism was proposed. As shown in Figure 6, firstly the metal center in the bifunctional catalyst activates the oxygen in the epoxide, then the halogen ion attacks nucleophilically the less site-resistant carbon of the epoxide, prompting ring opening of the epoxide to form a metal alcoholic salt intermediate, at which point carbon dioxide is inserted into the metal alcohol salt intermediate to form a metal carboxylate intermediate, and finally the product cyclic carbonate is generated through intramolecular cyclization. This mechanism suggests that the Lewis acid centers and Lewis base centers play a synergistic role in the cycloaddition reaction and are therefore essential in the catalytic system.

## 4. Conclusions

In this study, a series of bifunctional niobium complexes were synthesized and characterized by NMR, FTIR spectroscopy and elemental analysis. These catalysts can catalyze the formation of cyclic carbonates from epoxides and CO_2_ with high efficiency and selectivity in the absence of solvents and without co-catalysts. By systematic investigation, the optimum reaction conditions were screened as: reaction temperature of 100 °C, carbon dioxide pressure of 1 MPa, reaction time of 2 h and catalyst to epoxide ratio of 1:100. The substrate suitability of the catalysts was studied and the results showed that the catalysts were able to catalyze the cycloaddition of a wide range of epoxides with CO_2_ under both high and atmospheric conditions with high selectivity and good to excellent yields. Furthermore, the catalysts showed good recyclability via simple filtration and can be reused for at least five times without obvious loss of catalytic activity and selectivity. A kinetic study of bifunctional niobium complexes containing different halogen ions (3a(Cl^−^), 3b(Br^−^), 3c(I^−^)) was carried out and the order of apparent activation energies is 3a (96.2 kJ/mol) > 3b (68.2 kJ/mol) > 3c (37.4 kJ/mol). Finally, a proposed mechanism was given out based on kinetic study and the literature.

## Figures and Tables

**Table 1:** The effect of catalyst type on yield **^a^**.

Entry	Catalyst	PC yield (%) ^c^	Selectivity (%)
1	-	-	-
2	2a	78	99
3	2b	83	99
4	2c	89	99
5	2d	75	99
6	2e	73	99
7	3a	96	99
8	3b	97	99
9	3c	99	99
10	3d	49	99
11	3e	32	99
12	3f	13	98
13 ^b^	3a	76	99
14 ^b^	3b	78	99
15 ^b^	3c	79	99

^a^ Reaction conditions: propylene oxide (5 mL, 0.0714 mol), catalyst (0.714 mmol), temperature: 100 °C, CO_2_ pressure: 1 MPa, time: 2 h. ^b^ time: 1 h. ^c^ Yield and selectivity of the products were examined by ^1^H NMR (CDCl_3_, 400 MHz).

**Table 2:** Reaction of carbon dioxide with other epoxides catalyzed by bifunctional niobium complexes.

Entry	Epoxides	Product	Condition A ^a^		Condition B ^b^	
			Time (h)	Yield (%)	Time (h)	Yield (%)
1	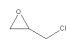	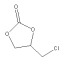	2	100	14	99.0
2	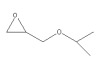	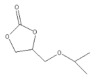	2	96.4	24	49.3
3	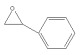	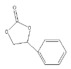	2	90.9	24	98.4
4	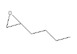	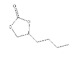	2	74.8	24	89.1
5	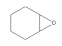	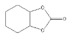	12	41.7	24	10.0
6	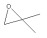	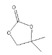	12	69.9	-	-
7	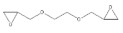	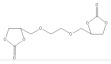	2	87.1	24	48.9
8	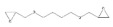	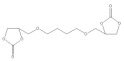	2	91.3	24	91.5
9	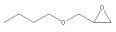	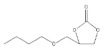	2	81.1	33	94.8
10	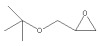	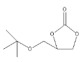	2	80.5	36	93.1
11	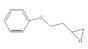	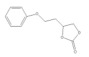	2	99.9	11	96.8
12	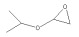	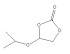	2	96.4	36	94.3

^a^ Reaction conditions: molar ratio of epoxy alkane (5 mL) to catalyst 1:100, 100 °C, 1 MPa, 2 h. ^b^ Reaction conditions: molar ratio of epoxy alkane (5 mL) to catalyst dosage 1:100, 100 °C, 0.1 MPa. The yield and selectivity of the products were detected by ^1^H NMR (CDCl_3_, 400 MHz) and all selectivities are >99%.

## Data Availability

The data presented in this study are openly available in MDPI.

## Supplementary Materials

The following supplementary materials can be downloaded at: https://www.mdpi.com/article/10.3390/ma16093531/s1, Table S1: Characterization of intermediates. Table S2.1: The relationship between the yield and time of the reaction of CO_2_ and *n*-butyl glycidyl ether catalyzed by compound 3a under 353 K atmospheric conditions. Table S2.2: The relationship between the yield and time of the reaction of CO_2_ and *n*-butyl glycidyl ether catalyzed by compound 3a under 373 K atmospheric conditions. Table S2.3: The relationship between the yield and time of the reaction of CO_2_ and *n*-butyl glycidyl ether catalyzed by compound 3a under 393 K atmospheric conditions. Table S2.4: The relationship between the yield and time of the reaction of CO_2_ and *n*-butyl glycidyl ether catalyzed by compound 3a under 413 K atmospheric conditions. Figure S1: NMR spectra of the substances synthesized in this work. Figure S2.1: The relationship between the ln(1 − x) and time *t* of the bifunctional niobium complex 3a catalyzed by the cycloaddition reaction at 353 K-413 K. Table S2.5: The relationship between the yield and time of the reaction of CO_2_ and n-butyl glycidyl ether catalyzed by compound 3b under 353 K atmospheric conditions. Table S2.6: The relationship between the yield and time of the reaction of CO_2_ and *n*-butyl glycidyl ether catalyzed by compound 3b under 373 K atmospheric conditions. Table S2.7: The relationship between the yield and time of the reaction of CO_2_ and *n*-butyl glycidyl ether catalyzed by compound 3b under 393 K atmospheric conditions. Table S2.8: The relationship between the yield and time of the reaction of CO_2_ and *n*-butyl glycidyl ether catalyzed by compound 3b under 413 K atmospheric conditions. Figure S2.2: The relationship between the ln(1 − *x*) and time t of the bifunctional niobium complex 3b catalyzed by the cycloaddition reaction at 353 K–413 K. Table S2.9: The relationship between the yield and time of the reaction of CO_2_ and *n*-butyl glycidyl ether catalyzed by compound 3c under 353 K atmospheric conditions. Table S2.10: The relationship between the yield and time of the reaction of CO_2_ and *n*-butyl glycidyl ether catalyzed by compound 3c under 373 K atmospheric conditions. Table S2.11: The relationship between the yield and time of the reaction of CO_2_ and *n*-butyl glycidyl ether catalyzed by compound 3c under 393 K atmospheric conditions. Figure S2.3: The relationship between the ln(1 − *x*) and time *t* of the bifunctional niobium complex 3c catalyzed by the cycloaddition reaction at 353 K–393 K. Figure S2.4: Arrhenius linear relationship between carbon dioxide and *n*-butyl glycidyl ether catalyzed by different halogen bifunctional niobium complexes.
