# Supplementary material for: Solvent- and Co-Catalyst-Free Cycloaddition of Carbon Dioxide and Epoxides Catalyzed by Recyclable Bifunctional Niobium Complexes
Source: Materials (Basel). 2023 May 4;16(9):3531. doi: 10.3390/ma16093531 (PMC10179855; doi:10.3390/ma16093531)
Supplement: Supplementary file 1 [file materials-16-03531-s001.zip › materials-2320592-supplementary.pdf]

Supplementary Materials

Solvent- and Co-Catalyst-Free Cycloaddition of Carbon Dioxide and Epoxides Catalyzed by Recyclable Bifunctional Niobium Complexes

Qin Wen [1,†], Xuexin Yuan [1,†], Qiqi Zhou [1], Hai-Jian Yang [1,*], Qingqing Jiang [1], Juncheng Hu [1] and Cun-Yue Guo [2,*]

1 Key Laboratory of Catalysis and Energy Materials Chemistry of Ministry of Education,
Hubei Key Laboratory of Catalysis and Materials Science, College of Chemistry and Materials Science, South-Central Minzu University, Wuhan 430074, China; wenqindengzihan@sina.com (Q.W.); yuanxx0223@sina.com (X.Y.); zhouqiqi98814@sina.com (Q.Z.); qqjiang@mail.scuec.edu.cn (Q.J.); jchu@mail.scuec.edu.cn (J.H.)

2 School of Chemical Sciences, University of Chinese Academy of Sciences, Beijing 100049, China

* Correspondence: yanghaijian@vip.sina.com (H.-J.Y.); cyguo@ucas.ac.cn (C.-Y.G.)

† These authors contributed equally to this work.

Citation: Wen, Q.; Yuan, X.; Zhou, Q.; Yang, H.-J.; Jiang, Q.; Hu, J.; Guo, C.-Y. Solvent- and Co-Catalyst-Free Cycloaddition of Carbon Dioxide and Epoxides Catalyzed by Recyclable Bifunctional Niobium Complexes. *Materials* **2023**, *16*, 3531. https://doi.org/10.3390/ma16093531

Academic Editors: Ovidiu Oprea and Paula Teixeira

Received: 18 March 2023
Revised: 1 May 2023
Accepted: 3 May 2023
Published: 4 May 2023

Copyright: © 2023 by the authors. Licensee MDPI, Basel, Switzerland. This article is an open access article distributed under the terms and conditions of the Creative Commons Attribution (CC BY) license (https://creativecommons.org/licenses/by/4.0/).

Provenance and Mass Fraction Purity of the Materials

Materials	Mass fraction	CAS	Provenance
2-Hydroxybenzaldehyde	0.98	90-02-8	Shanghai Aladdin Bio-Chem Technology Co., Ltd.
Paraformaldehyde	AR	30525-89-4	Shanghai Aladdin Bio-Chem Technology Co., Ltd.
Hydrochloric acid	AR	7647-01-0	Sinopharm Chemical Reagent Co., Ltd
Hydrogen bromide	AR	10035-10-6	Sinopharm Chemical Reagent Co., Ltd
Hydriodic acid	AR	10034-85-2	Beijing J&K Scientific Ltd.
1-Methylimidazole	0.98	616-47-7	Shanghai Darui Finechemical Co., Ltd.
1-Ethylimidazole	0.98	7098-07-9	Shanghai TCI Development Co., Ltd.
1-Butylimidazole	0.99	4316-42-1	Beijing J&K Scientific Ltd.
2-Aminophenol	0.98	95-55-6	Shanghai Darui Finechemical Co., Ltd.
Ethanol	AR	64-17-5	Sinopharm Chemical Reagent Co., Ltd
Niobium chloride	0.99	10026-12-7	USA Strem Chemicals, Inc.
Epichlorohydrin	0.99	106-89-8	Beijing J&K Scientific., Ltd.
Glycidyl isopropyl ether	0.99	4016-14-2	Shanghai TCI Development Co., Ltd.
Styrene oxide	0.98	96-09-3	Shanghai Darui FinechemicalCo., Ltd.
1,2-Epoxyhexane	0.96	1436-34-6	Shanghai TCI Development Co., Ltd.
Cyclohexene oxide	0.98	286-20-4	Shanghai Darui FinechemicalCo., Ltd.

Isobutylene oxide	0.98	558-30-5	Beijing J&K Scientific., Ltd.
Ethylene glycol diglycidyl ether	0.99	2224-15-9	Beijing J&K Scientific., Ltd.
1,4-Butanediol diglycidyl ether	0.99	2425-79-8	Shanghai Darui FinechemicalCo., Ltd.
Butyl glycidyl ether	0.98	2426-08-6	Shanghai Darui FinechemicalCo., Ltd.
Tert-butyl glycidyl erher	0.96	7665-72-7	Beijing J&K Scientific., Ltd.
2-(2-phenoxyethyl)oxirane	0.96	21746-93-0	Beijing J&K Scientific., Ltd.
2-isopropoxyoxirane	0.96	294210-20-1	Beijing J&K Scientific., Ltd.

Characterization of intermediates

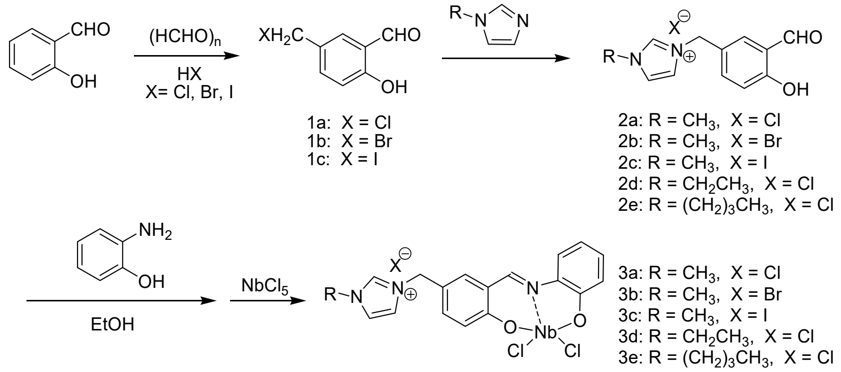

Table S1 Characterization of intermediates

2.1.1 Synthesis of compounds 1

13.5 g of paraformaldehyde was weighed into a two-necked flask equipped with stir bar and 150 mL of HX (Cl, Br, I) and 26 mL of salicylaldehyde were added under the protection of N_2 and stirred at room temperature for three days to produce a large amount of pink solid. The pink solid was filtered under reduced pressure and washed with a large amount of saturated sodium bicarbonate solution. After being dried under vacuum, it was dissolved in dichloromethane and dried overnight with anhydrous magnesium sulfate, filtered, and the dichloromethane was removed by spin evaporation, then the crude product was recrystallized with petroleum ether and dried under vacuum to give a white solid. The method has been reported in many papers, so only the ^{1}H NMR spectrum data are tested to confirm the structures of compounds (please refer to the Supplementary Material).

1a: white solid, yield 60%, melting point 68-70 °C.^{1}H NMR (400 MHz, DMSO) δ 10.92 (s, 1H), 10.27 (d, J = 2.7 Hz, 1H), 7.72 (d, J = 2.3 Hz, 1H), 7.58 (m, 1H), 7.01 (t, J = 8.9 Hz, 1H), 4.75 (s 2H).

1b: white solid, yield 67%, melting point 70-72°C.^{1}H NMR (400 MHz, DMSO) δ 10.92 (s, 1H), 10.26 (s, 1H), 7.60 (s, 1H), 7.48 - 7.43 (m, 1H), 6.97 (d, J = 8.5 Hz, 1H), 4.42 (s , 2H).

1c: white solid, yield 50%, melting point 94-96 °C.^{1}H NMR (400 MHz, DMSO) δ 11.24 (s, 1H), 10.26 (d, J = 7.1 Hz, 1H), 7.60 (d, J = 2.0 Hz, 1H), 7.52 - 7.42 (m, 1H), 6.97 (m, 1H), 4.43 (d, J = 8.2 Hz, 2H).

2.1.2 Synthesis of compound 2

Referring to literature [22], 0.025 mol of compound 1 was weighed, dissolved in 20 mL of tetrahydrofuran, and 0.0375 mol of imidazoles was slowly added dropwise at room temperature and stirred for 2 h. The crude product obtained was recrystallized with methanol and tetrahydrofuran to obtain purified compound 2.

2a: pale yellow solid, yield 65%, melting point 81-85 °C.^{1}H NMR (400 MHz, D_2O) δ 9.91 (d, J = 10.0 Hz, 1H), 8.72 (s, 1H), 7.71 (d, J = 3.4 Hz, 1H), 7.54 (d, J = 8.7 Hz, 1H), 7.41 (d, J = 7.3 Hz, 2H). 6.98 (d, J = 8.6 Hz, 1H), 5.39 (d, J = 18.2 Hz, 2H), 3.86 (s, 3H).

2b: pale yellow solid, yield 42%, melting point 95-99 °C.^{1}H NMR (400 MHz, D_2O) δ 9.64 (d, J = 9.7 Hz, 1H), 8.32 (s, 1H), 7.41 (d, J = 4.2 Hz, 1H), 7.21 (d, J = 9.5 Hz, 1H), 7.06 (d, J = 5.4 Hz, 2H). 6.65 (d, J = 7.5 Hz, 1H), 5.07 (d, J = 19.6 Hz, 2H), 3.46 (s, 3H).

2c: pale yellow solid, 29% yield, melting point 96-100°C.^{1}H NMR (400 MHz, D_2O) δ 9.44 (d, J = 8.6 Hz, 1H), 8.01 (s, 1H), 7.15 (d, J = 2.8 Hz, 1H), 6.93 (d, J = 7.8 Hz, 1H), 6.82 (d, J = 4.9 Hz, 2H). 6.74 (d, J = 7.9 Hz, 1H), 4.86 (d, J = 17.8 Hz, 2H), 3.25 (s, 3H).

2d: pale yellow solid, 27% yield, melting point 103-107 °C.^{1}H NMR (400 MHz, DMSO) δ 12.31 (s, 1H), 10.56 (s, 1H), 7.87 - 7.64 (m, 3H), 7.57 (d, J = 7.3, 1.8 Hz, 1H), 7.35 (d, J = 9.4 Hz, 1H), 5.49 (s, 2H), 3.94 - 3.83 (m, 2H), 2.63 - 2.42 (m, 3H).

2e: pale yellow solid, yield 21%, melting point 105-109 °C.^{1}H NMR (400 MHz, $CDCl_3$) δ 11.36 - 11.18 (m, 1H), 10.24 (s, 1H), 9.76 (s, 1H), 7.81 (s, 1H), 7.67 (s, 1H), 7.51 (d, J = 7.4, 2.4 Hz, 1H), 7.18 (s, 1H), 6.85 (d, J = 7.1 Hz, 1H), 5.46 (d, J = 4.7 Hz, 2H), 4.07 (d, J = 15.6, 7.3 Hz, 2H), 1.71 - 1.43 (m, 2H), 1.21 - 1.04 (m, 2H), 0.52 (t, J = 6.1 Hz, 3H).

Reaction kinetics study

We have also studied the kinetics of the bifunctional niobium complex catalyzed by the cycloaddition reaction of carbon dioxide. The model reaction is the reaction of n-butyl glycidyl ether (BGE) and CO_2 under normal pressure. The kinetic behavior of the bifunctional niobium complex containing different halogen ions in the reaction time interval of 2 h to 8 h. Because the iodine catalyst is too active, the reaction is close to completion in 2 h at 413K. Data, so this interval is omitted. Known in the literature [27], the general rate formula is shown in formula (2.1), which represents the cycloaddition reaction of CO_2 and BGE. In this formula, because of the large amount of CO_2 in the reaction process, the concentration of the catalyst can be used as a fixed value, so the formula (2.1) is simplified to obtain the formula (2.2). Assuming that the reaction is a first-order reaction, $\alpha = 1$, the yield of the cyclic carbonate produced by the reaction time t is x, and then the equivalent transformation equation (2.2) can be transformed into equations (2.3) and (2.4). The experimental results are consistent with the formula (2.4), which shows that the reaction rate and the concentration of BGE have a realizable relationship, and further shows that the reaction rate in this reaction is a first-order reaction to the concentration of BGE. The following figure S2.4 shows the linear relationship of the bifunctional niobium complex containing different halogen ions. According to the Arrhenius empirical formula, the activation energy of the reaction is calculated from the formula (2.5). The activation energy sequence is 3a > 3b > 3c is consistent with the conclusion: the greater the catalyst activity, the smaller the activation energy is. The results are agreement with the leaving ability of the contained halogen ions, $Cl^- < Br^- < I^-$.

$$r = dx/dt = k''[BGE]^{\alpha}[CO_2]^{\beta}[Cat.]^{\gamma} \tag{S2.1}$$

$$r = dx/dt = k'[BGE]^{\alpha} \tag{S2.2}$$

$$r = dx/dt = k[BGE] = k(1-x) \tag{S2.3}$$

$$\ln(1-x) = -kt + C \tag{S2.4}$$

$$\ln k = -E_a/RT + C \tag{S2.5}$$

Table S2.1 The relationship between the yield and time of the reaction of CO_2 and *n*-butyl glycidyl ether catalyzed by compound 3a under 353 K atmospheric conditions

Entry	Time (h)	Yield (%)^{a,b}	Selectivity (%)^{b}
1	2	1.0	> 99
2	4	2.2	> 99
3	6	3.3	> 99
4	8	4.3	> 99

a: Reaction conditions: n-butyl glycidyl ether (5 mL) to catalyst dosage molar ratio 1:100, temperature: 80 °C, CO_2 pressure: 0.1 MPa.

b: Yield and selectivity of the products were examined by ^{1}H NMR ($CDCl_3$, 400 MHz).

Table S2.2 The relationship between the yield and time of the reaction of CO_2 and *n*-butyl

glycidyl ether catalyzed by compound 3a under 373 K atmospheric conditions

Entry	Time (h)	Yield (%)[a,b]	Selectivity (%)[b]
1	2	0	➢ 99
2	4	1.0	> 99
3	6	4.4	> 99
4	8	10.9	> 99

a: Reaction conditions: n-butyl glycidyl ether (5 mL) to catalyst dosage molar ratio 1:100, temperature: 100 ^oC, CO_2 pressure: 0.1 MPa.

b: Yield and selectivity of the products were examined by ^1H NMR ($CDCl_3$, 400 MHz).

Table S2.3 The relationship between the yield and time of the reaction of CO_2 and *n*-butyl glycidyl ether catalyzed by compound 3a under 393 K atmospheric conditions

Entry	Time (h)	Yield (%)[a,b]	Selectivity (%)[b]
1	2	11.1	> 99
2	4	31.7	> 99
3	6	48.9	> 99
4	8	65.0	> 99

a: Reaction conditions: n-butyl glycidyl ether (5 mL) to catalyst dosage molar ratio 1:100, temperature: 120 ^oC, CO_2 pressure: 0.1 MPa.

b: Yield and selectivity of the products were examined by 1H NMR ($CDCl_3$, 400 MHz).

Table S2.4 The relationship between the yield and time of the reaction of CO_2 and *n*-butyl glycidyl ether catalyzed by compound 3a under 413 K atmospheric conditions

Entry	Time (h)	Yield (%)[a,b]	Selectivity (%)[b]
1	2	37.4	>99
2	4	71.2	>99
3	6	92.9	>99
4	8	97.2	>99

a: Reaction conditions: *n*-butyl glycidyl ether (5 mL) to catalyst dosage molar ratio 1:100, temperature: 140 ^oC, CO_2 pressure: 0.1 MPa.

b: Yield and selectivity of the products were examined by ^1H NMR ($CDCl_3$, 400 MHz).

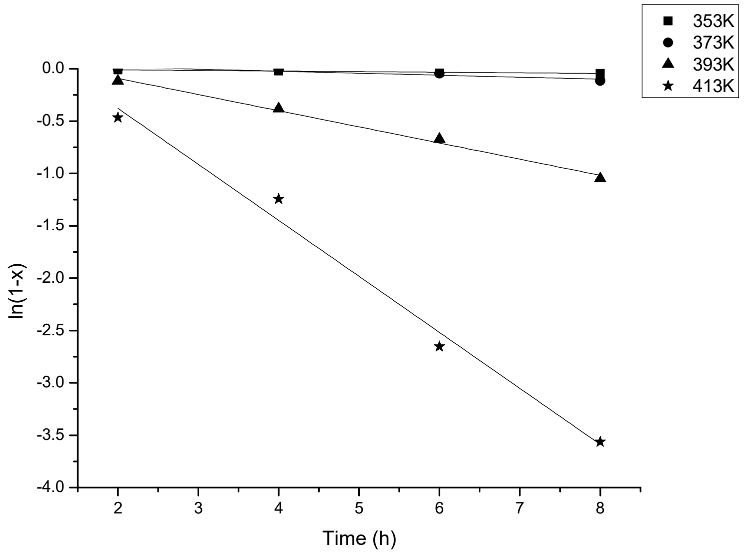

Figure S2.1 The relationship between the ln(1-*x*) and time *t* of the bifunctional niobium complex 3a catalyzed by the cycloaddition reaction at 353 K-413 K

Table S2.5 The relationship between the yield and time of the reaction of CO_2 and *n*-butyl glycidyl ether catalyzed by compound 3b under 353 K atmospheric conditions

Entry	Time (h)	Yield (%)[a,b]	Selectivity (%)[b]
1	2	0	> 99
2	4	0	> 99
3	6	3.7	> 99
4	8	9.4	> 99

a: Reaction conditions: *n*-butyl glycidyl ether (5 mL) to catalyst dosage molar ratio 1:100, temperature: 80 °C, CO_2 pressure: 0.1 MPa.

b: Yield and selectivity of the products were examined by ^{1}H NMR ($CDCl_3$, 400 MHz).

Table S2.6 The relationship between the yield and time of the reaction of CO_2 and *n*-butyl glycidyl ether catalyzed by compound 3b under 373 K atmospheric conditions

Entry	Time (h)	Yield (%)[a,b]	Selectivity (%)[b]
1	2	8.4	> 99
2	4	13.0	> 99
3	6	38.0	> 99
4	8	50.0	> 99

a: Reaction conditions: *n*-butyl glycidyl ether (5 mL) to catalyst dosage molar ratio 1:100, temperature: 100 °C, CO_2 pressure: 0.1 MPa.

b: Yield and selectivity of the products were examined by ^{1}H NMR ($CDCl_3$, 400 MHz).

Table S2.7 The relationship between the yield and time of the reaction of CO_2 and *n*-butyl glycidyl ether catalyzed by compound 3b under 393 K atmospheric conditions

Entry	Time (h)	Yield (%)[a,b]	Selectivity (%)[b]
1	2	56.2	> 99
2	4	77.1	> 99
3	6	83.3	> 99
4	8	92.0	> 99

a: Reaction conditions: *n*-butyl glycidyl ether (5 mL) to catalyst dosage molar ratio 1:100, temperature: 120 °C, CO_2 pressure: 0.1 MPa.

b: Yield and selectivity of the products were examined by ^{1}H NMR ($CDCl_3$, 400 MHz).

Table S2.8 The relationship between the yield and time of the reaction of CO_2 and *n*-butyl glycidyl ether catalyzed by compound 3b under 413 K atmospheric conditions

Entry	Time (h)	Yield (%)[a,b]	Selectivity (%)[b]
1	2	80.0	> 99
2	4	91.0	> 99
3	6	97.0	> 99
4	8	99.0	> 99

a: Reaction conditions: n-butyl glycidyl ether (5 mL) to catalyst dosage molar ratio 1:100, temperature: 140 °C, CO_2 pressure: 0.1 MPa.

b: Yield and selectivity of the products were examined by ^{1}H NMR ($CDCl_3$, 400 MHz).

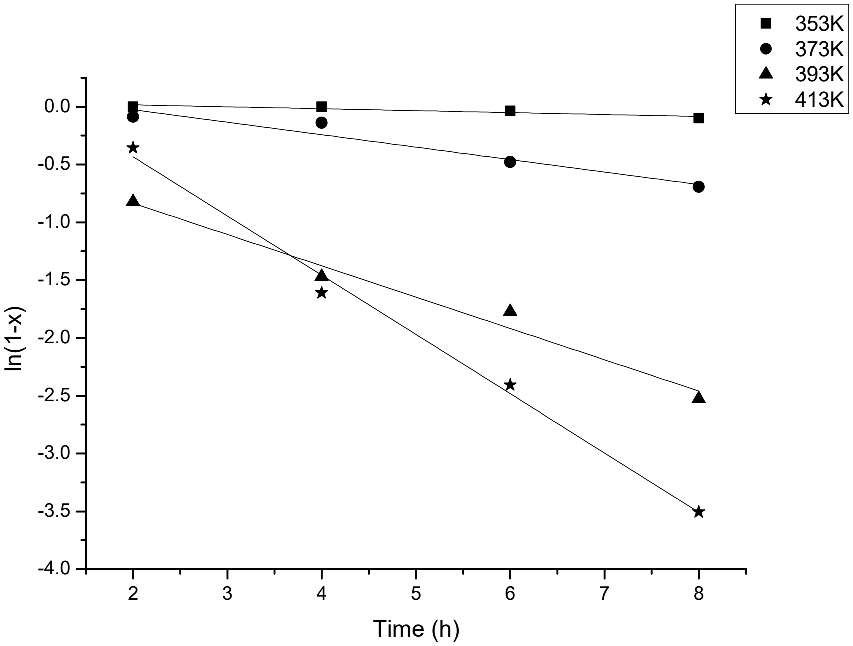

Figure S2.2 The relationship between the ln(1-x) and time t of the bifunctional niobium complex 3b catalyzed by the cycloaddition reaction at 353 K-413 K

Table S2.9 The relationship between the yield and time of the reaction of CO_2 and *n*-butyl glycidyl ether catalyzed by compound 3c under 353 K atmospheric conditions

Entry	Time (h)	Yield (%)[a,b]	Selectivity (%)[b]
1	2	5.5	> 99
2	4	26.4	> 99
3	6	50.4	> 99
4	8	63.6	> 99

a: Reaction conditions: *n*-butyl glycidyl ether (5 mL) to catalyst dosage molar ratio 1:100, temperature: 80 ^oC, CO_2 pressure: 0.1 MPa.

b: Yield and selectivity of the products were examined by ^1H NMR ($CDCl_3$, 400 MHz).

Table S2.10 The relationship between the yield and time of the reaction of CO_2 and *n*-butyl glycidyl ether catalyzed by compound 3c under 373 K atmospheric conditions

Entry	Time (h)	Yield (%)[b]	Selectivity (%)[b]
1	2	43.7	> 99
2	4	82.5	> 99
3	6	94.2	> 99
4	8	96.6	> 99

a: Reaction conditions: n-butyl glycidyl ether (5 mL) to catalyst dosage molar ratio 1:100, temperature: 100 ^oC, CO_2 pressure: 0.1 MPa.

b: Yield and selectivity of the products were examined by ^1H NMR ($CDCl_3$, 400 MHz).

Table S2.11 The relationship between the yield and time of the reaction of CO_2 and *n*-butyl glycidyl ether catalyzed by compound 3c under 393 K atmospheric conditions

Entry	Time (h)	Yield (%)[b]	Selectivity (%)[b]
1	2	61.1	> 99
2	4	90.8	> 99
3	6	96.6	> 99

a: Reaction conditions: *n*-butyl glycidyl ether (5 mL) to catalyst dosage molar ratio 1:100, temperature: 120 ^oC, CO_2 pressure: 0.1 MPa.

b: Yield and selectivity of the products were examined by ^1H NMR ($CDCl_3$, 400 MHz).

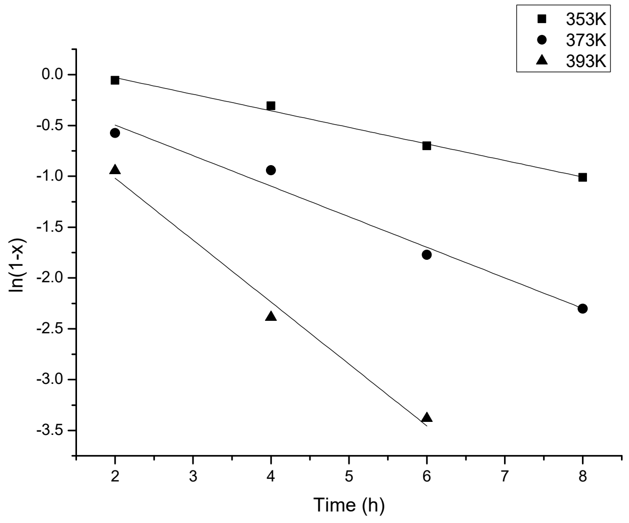

Figure S2.3 The relationship between the ln(1-x) and time t of the bifunctional niobium complex 3c catalyzed by the cycloaddition reaction at 353 K-393 K

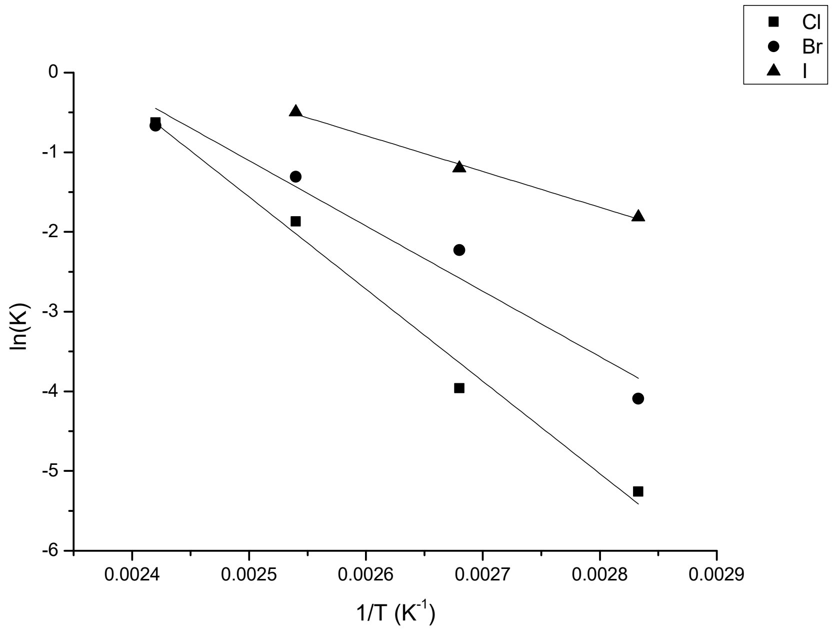

Figure S2.4 Arrhenius linear relationship between carbon dioxide and n-butyl glycidyl ether catalyzed by different halogen bifunctional niobium complexes

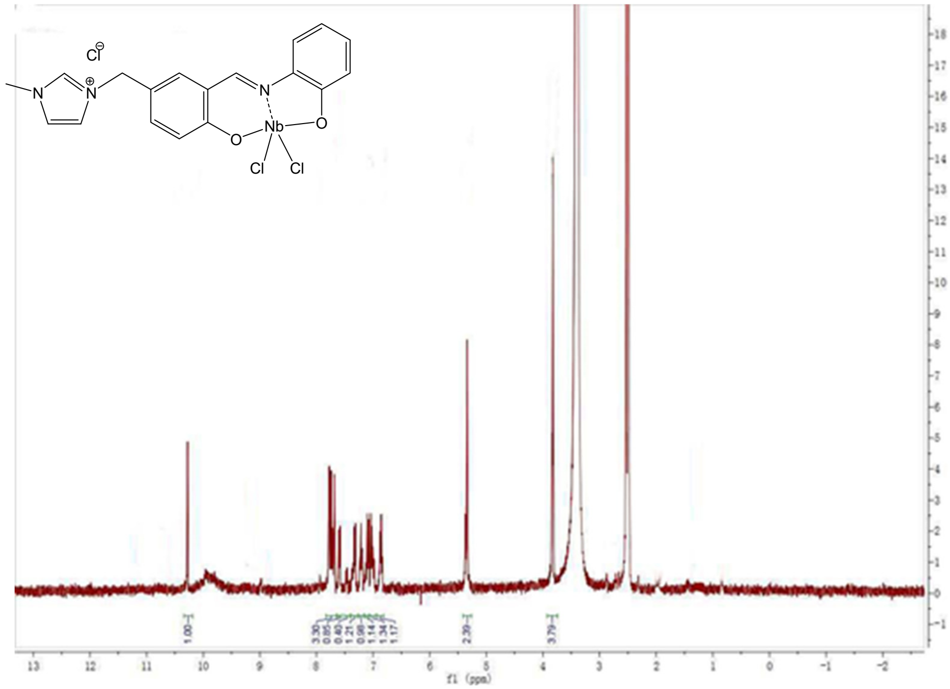
Cl⊖
N
N⊕
N
O
Nb
O
Cl
Cl
1.00
3.30
0.85
0.40
1.21
0.98
1.14
1.34
1.17
2.39
3.79
f1 (ppm)

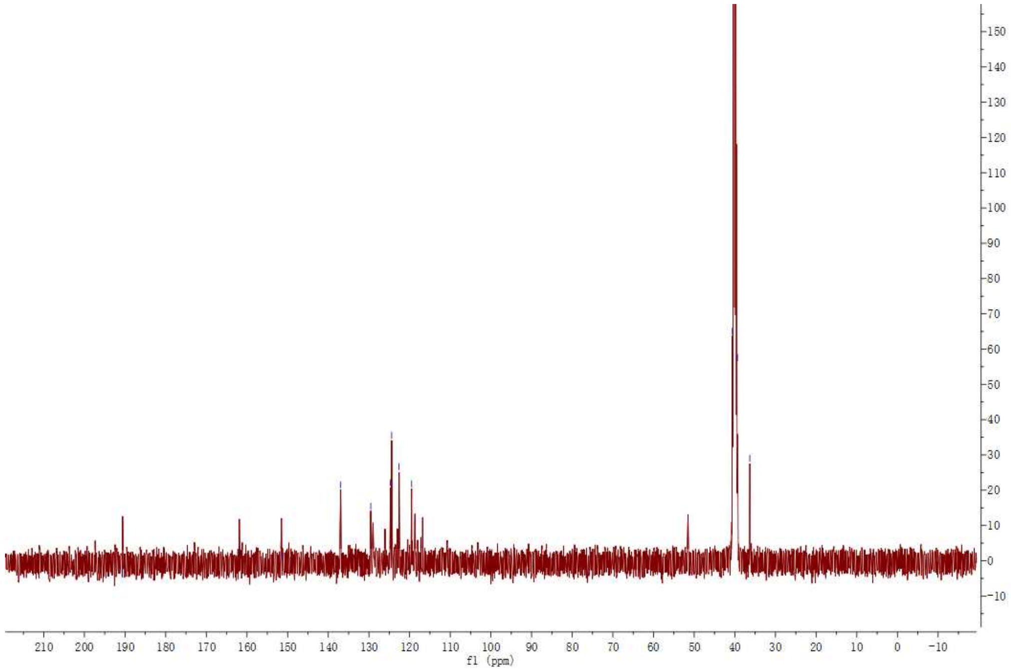
f1 (ppm)

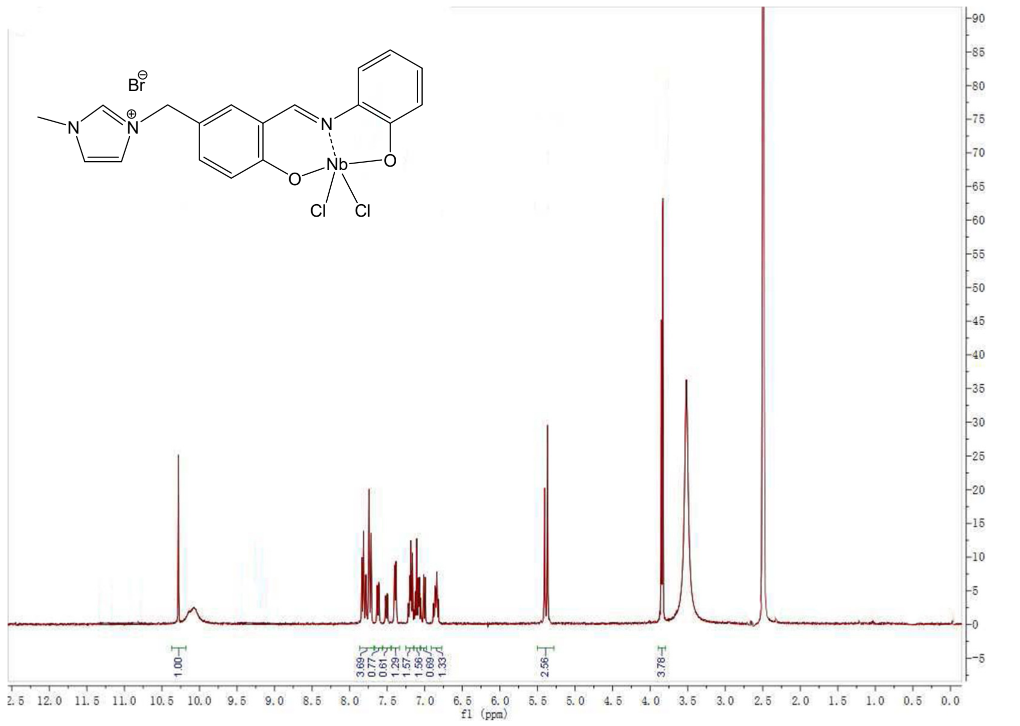

Br
N
N
Nb
O
O
Cl
Cl
f1 (ppm)

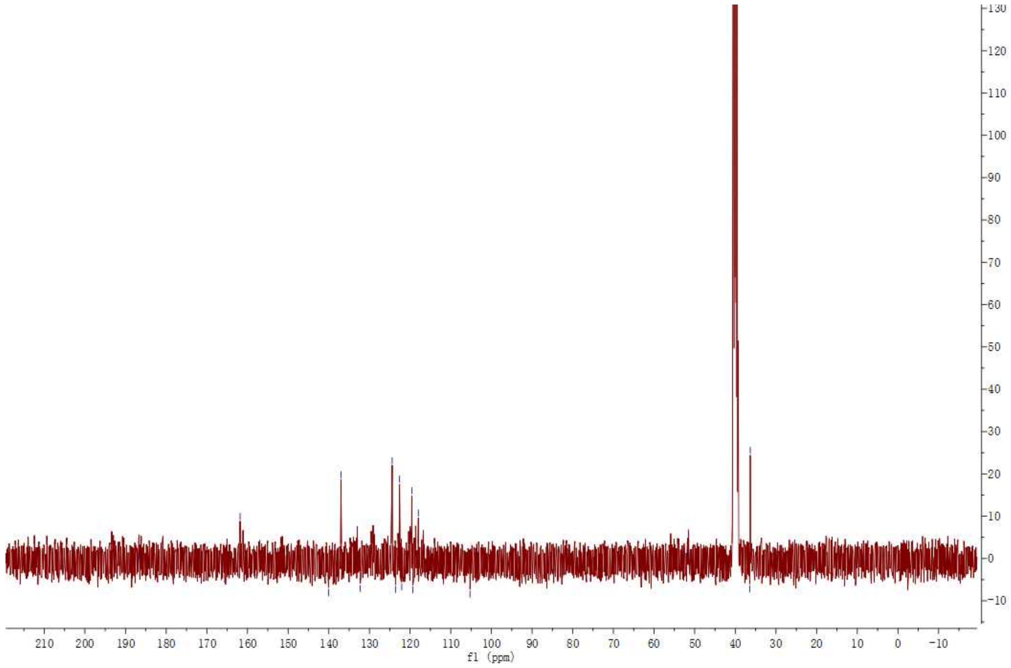

f1 (ppm)

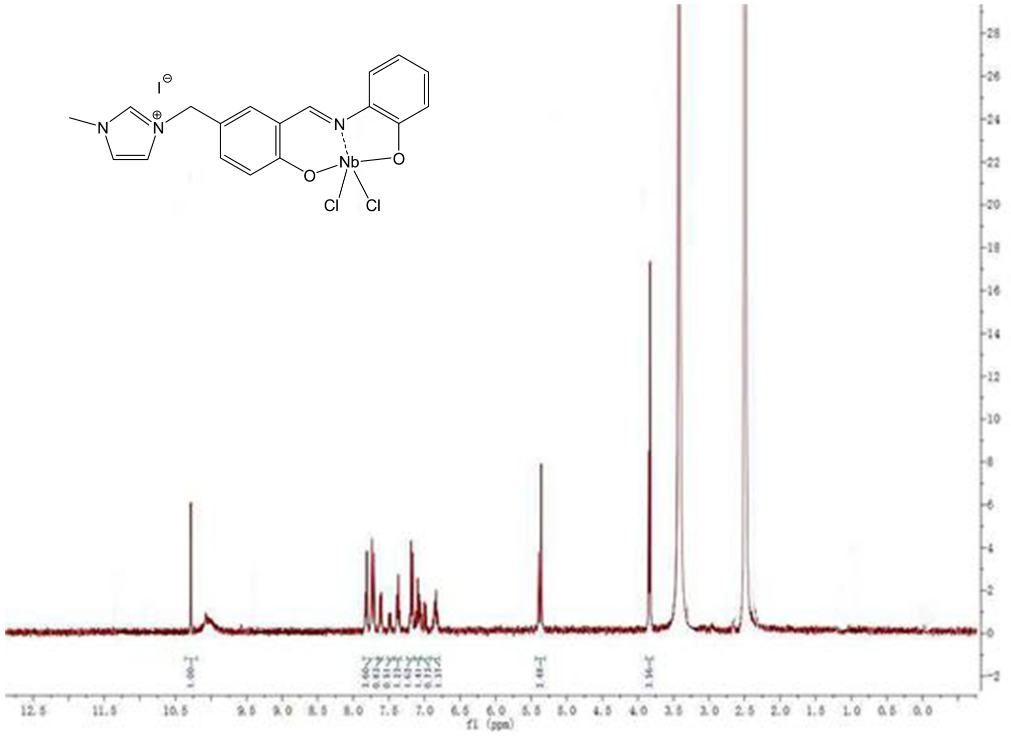

I
N
N
O
Nb
O
Cl
Cl
12.5 11.5 10.5 9.5 9.0 8.5 8.0 7.5 7.0 6.5 6.0 5.5 5.0 4.5 4.0 3.5 3.0 2.5 2.0 1.5 1.0 0.5 0.0
f1 (ppm)
28 26 24 22 20 18 16 14 12 10 8 6 4 2 0 -2

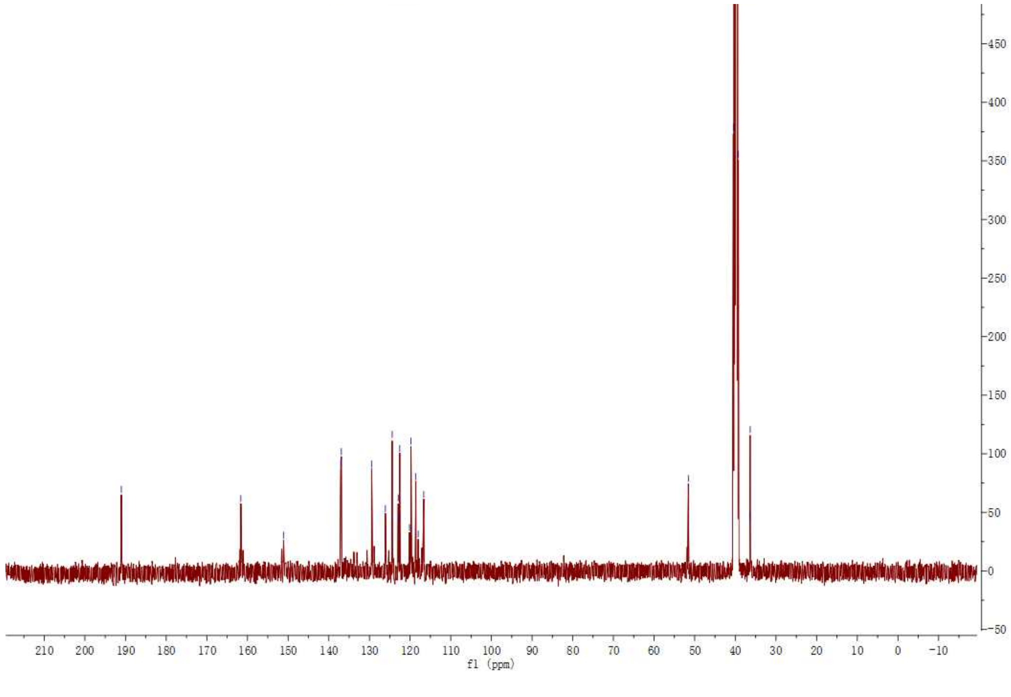

210 200 190 180 170 160 150 140 130 120 110 100 90 80 70 60 50 40 30 20 10 0 -10
f1 (ppm)
450 400 350 300 250 200 150 100 50 0 -50

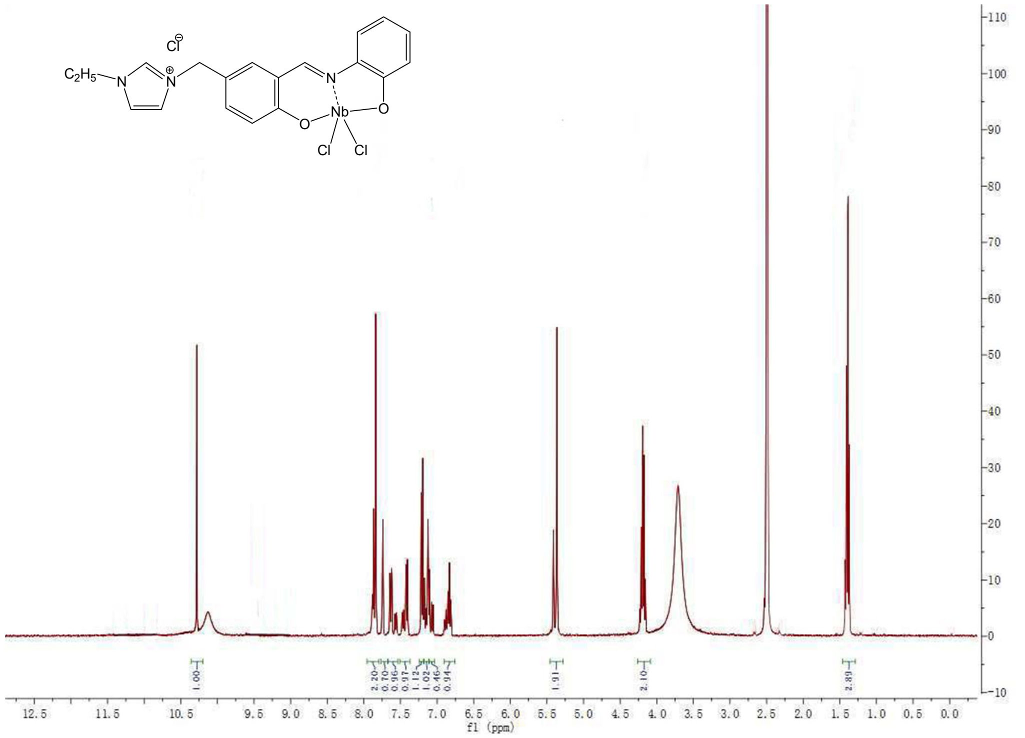

Cl⁻
C2H5
N
N+
N
Nb
O
O
Cl
Cl
1.00
2.20
0.70
0.96
0.97
1.12
1.02
0.46
0.94
1.91
2.10
2.89
f1 (ppm)

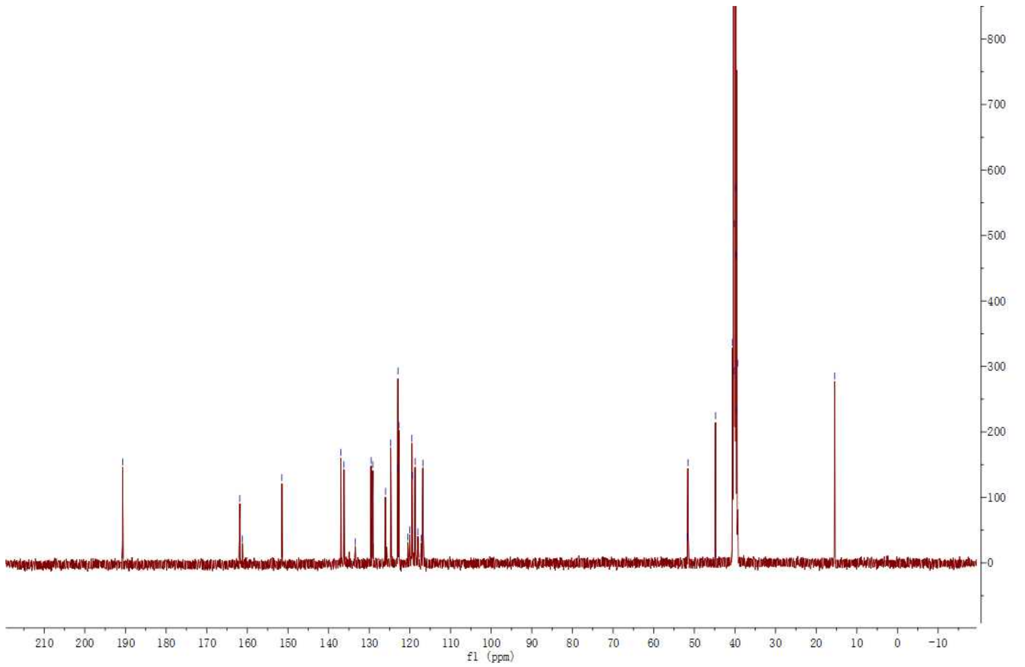

f1 (ppm)

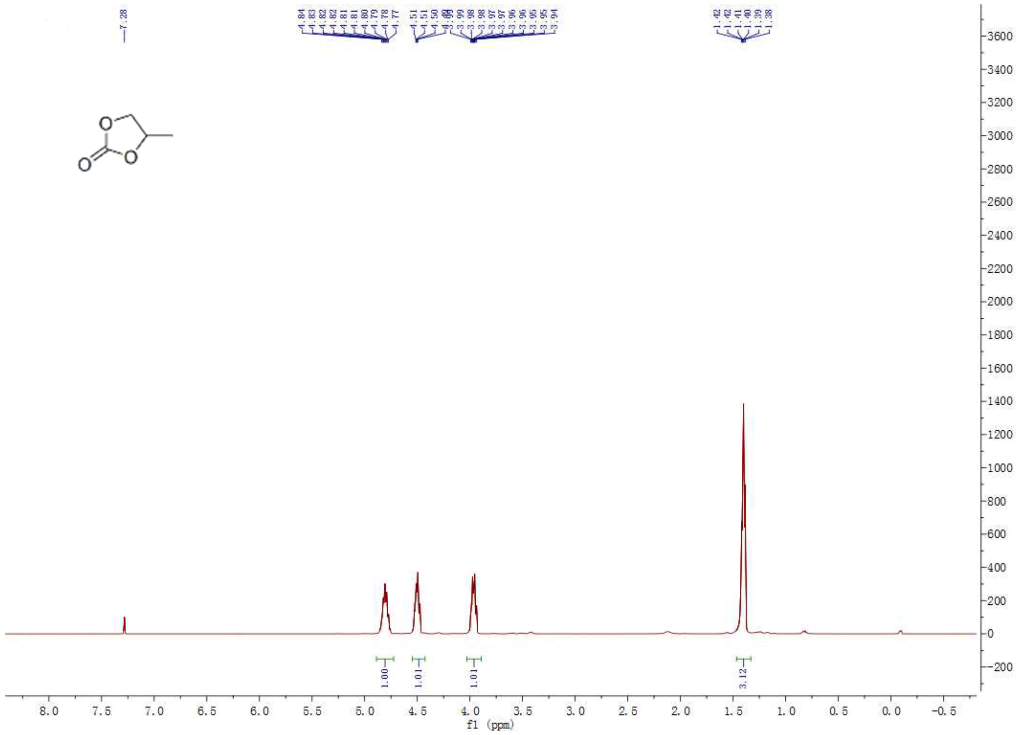
7.28
4.84 4.83 4.82 4.82 4.81 4.81 4.80 4.79 4.78 4.77
4.51 4.51 4.50
3.99 3.98 3.98 3.97 3.97 3.96 3.96 3.95 3.95 3.94
1.42 1.42 1.41 1.40 1.39 1.38
1.00
1.01
1.01
3.12
f1 (ppm)

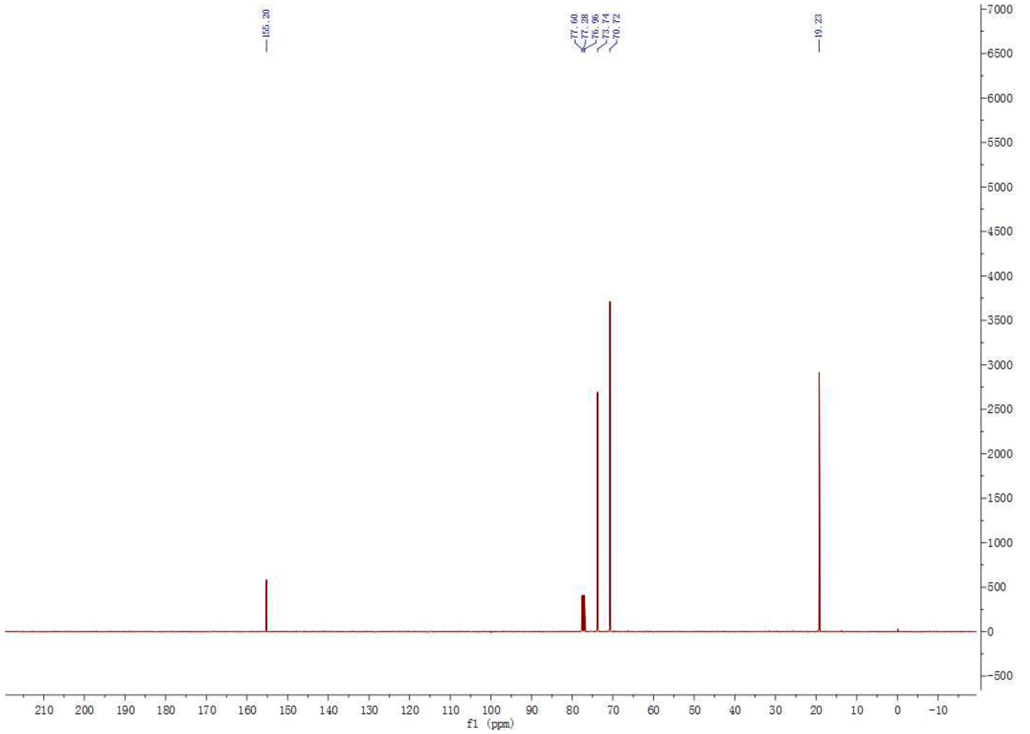
155.20
77.60 77.28 76.96 73.74 70.72
19.23
f1 (ppm)

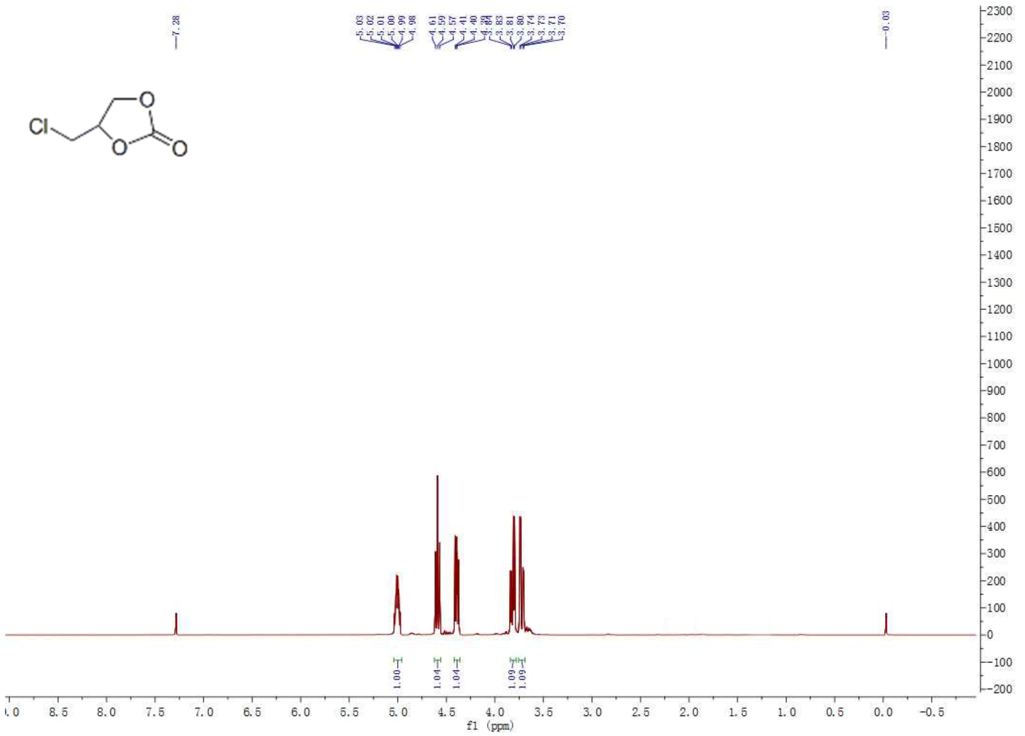

7.28
5.03
5.02
5.01
5.00
4.99
4.98
4.61
4.59
4.57
4.41
4.40
3.83
3.81
3.80
3.74
3.73
3.71
3.70
-0.03
Cl
O
O
O
1.00
1.04
1.04
1.09
1.09
f1 (ppm)

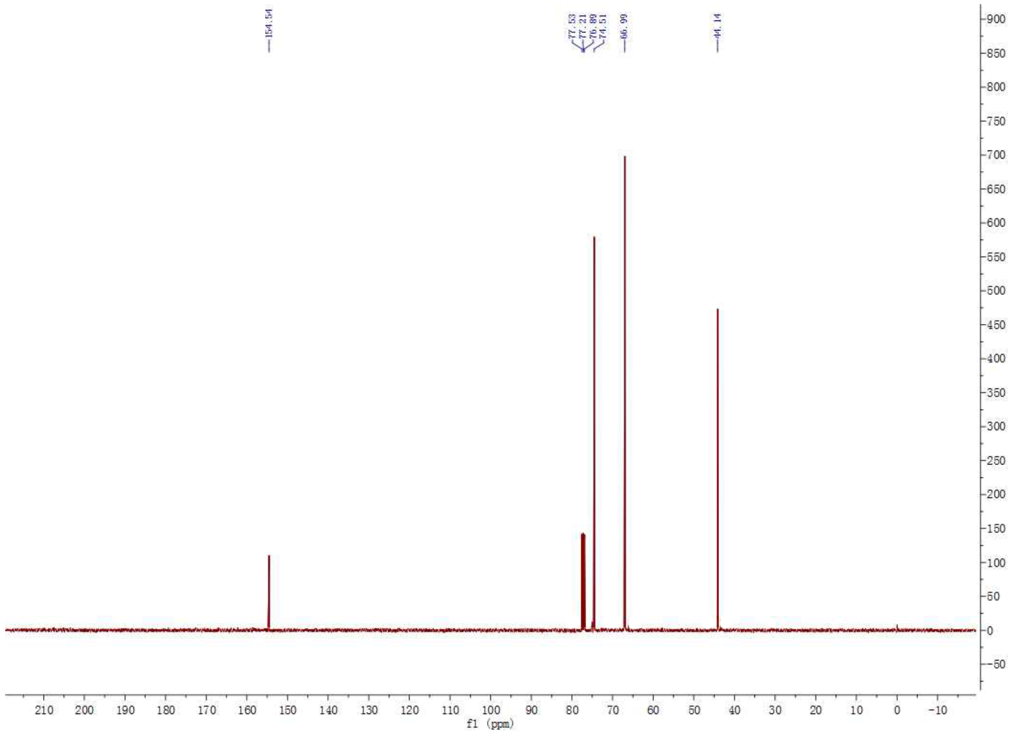

154.54
77.53
77.21
76.89
74.51
66.99
44.14
f1 (ppm)

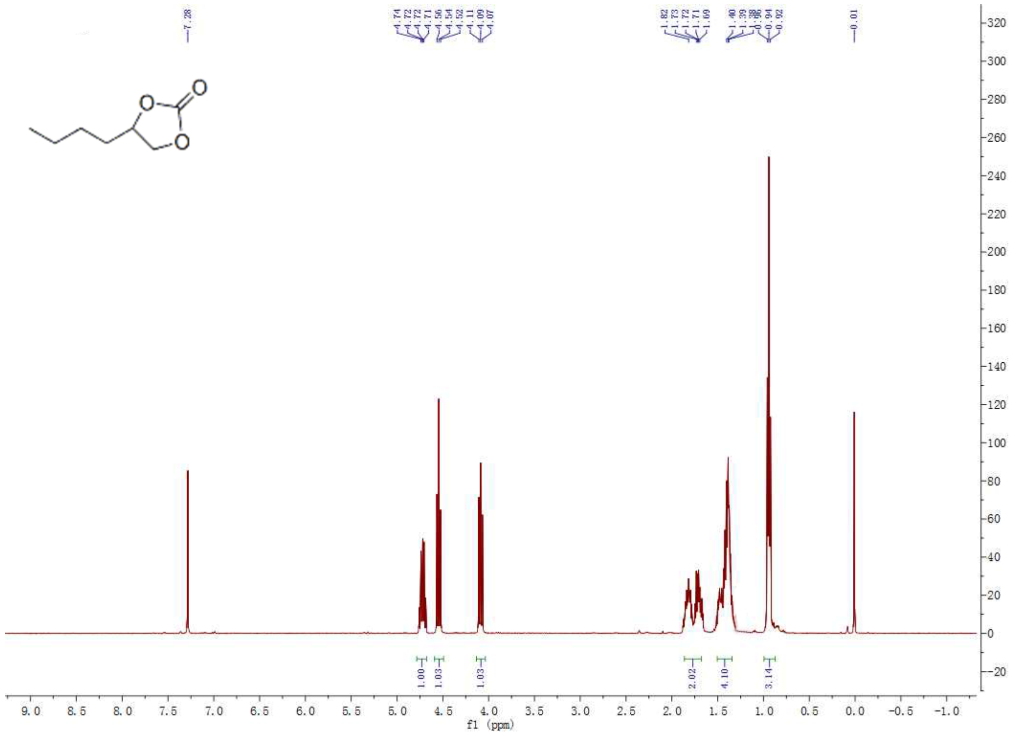

7.28
4.74
4.72
4.72
4.71
4.56
4.54
4.52
4.11
4.09
4.07
1.82
1.73
1.72
1.71
1.69
1.40
1.39
0.94
0.92
0.01
1.00
1.03
1.03
2.02
4.10
3.14
f1 (ppm)

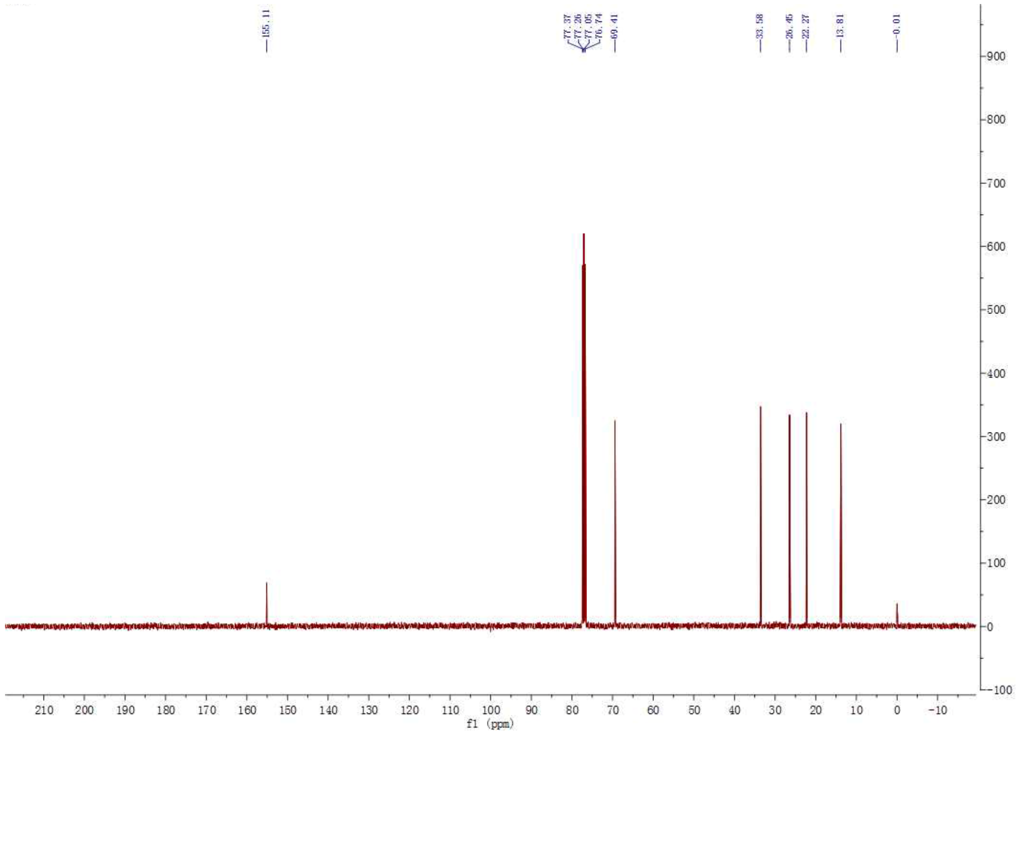

155.11
77.37
77.26
77.05
76.74
69.41
33.58
26.45
22.27
13.81
0.01
f1 (ppm)

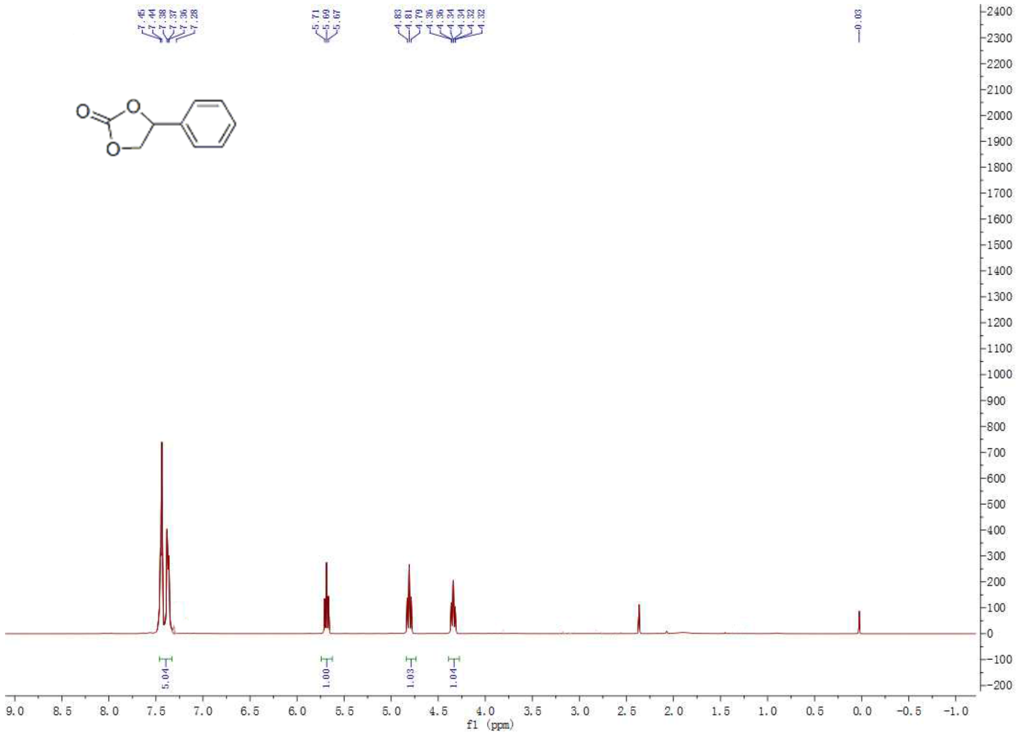
7.45
7.44
7.38
7.37
7.36
7.28
5.71
5.69
5.67
4.83
4.81
4.79
4.36
4.34
4.34
4.32
4.32
0.03
5.04
1.00
1.03
1.04
f1 (ppm)

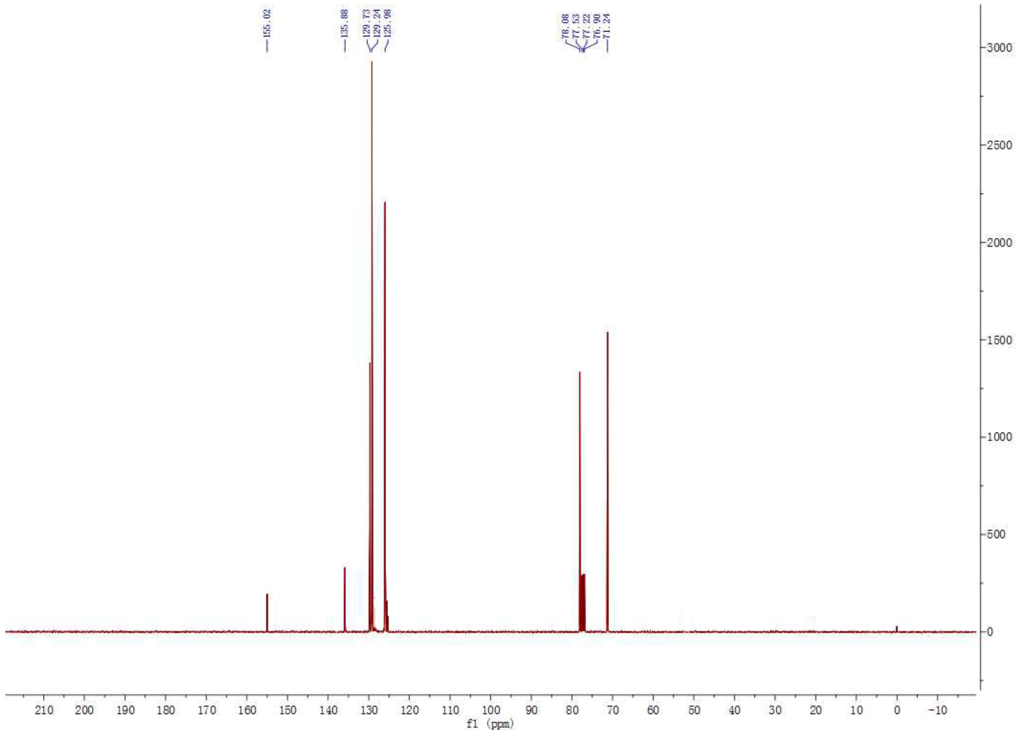
155.02
135.88
129.73
129.24
125.98
78.08
77.53
77.22
76.90
71.24
f1 (ppm)

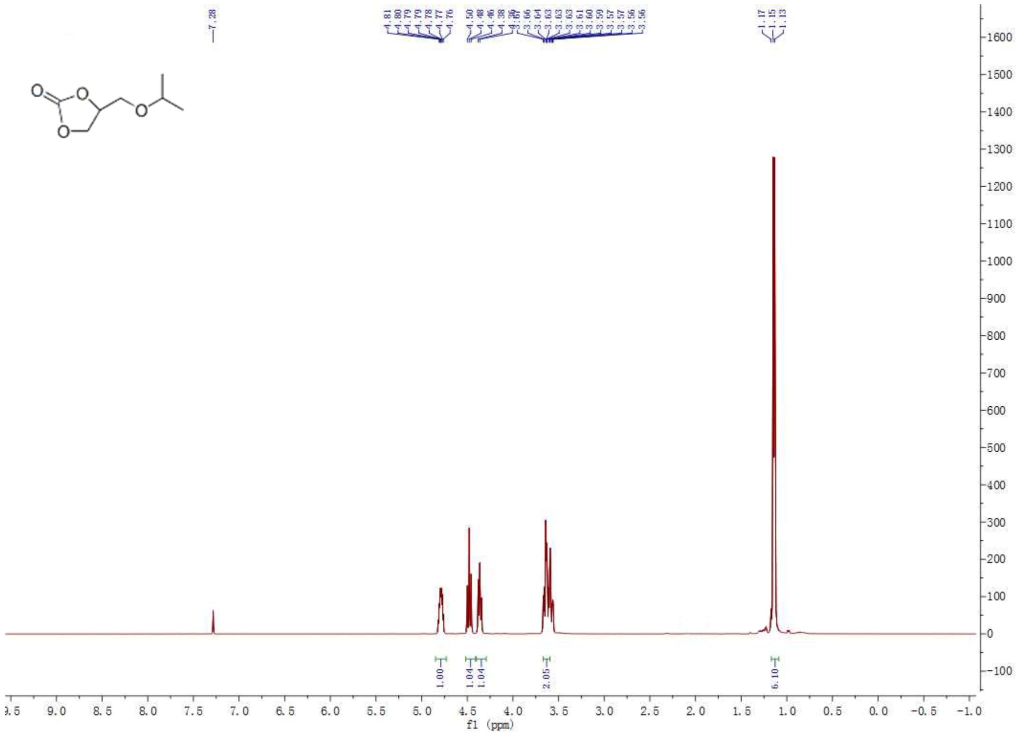

7.28
4.81 4.80 4.79 4.78 4.77 4.76
4.50 4.48 4.46 4.38
3.66 3.64 3.63 3.63 3.61 3.60 3.59 3.57 3.56 3.56
1.17 1.15 1.13
1.00
1.04
1.04
2.05
6.10
f1 (ppm)

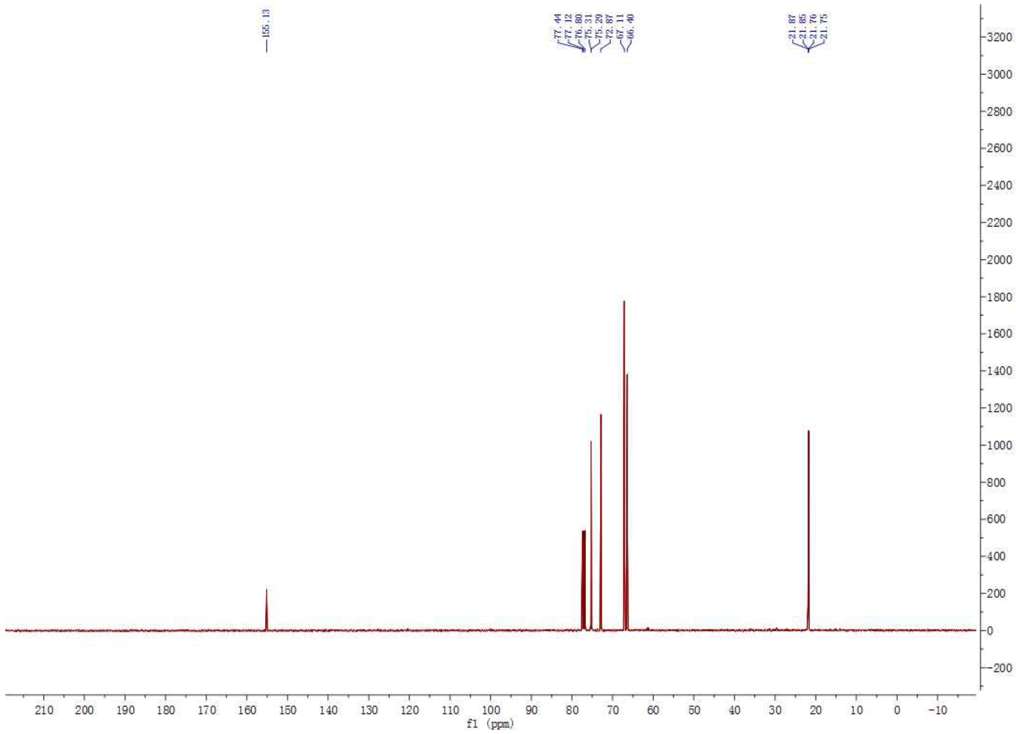

155.13
77.44 77.12 76.80 75.31 75.29 72.87 67.11 66.40
21.87 21.85 21.76 21.75
f1 (ppm)

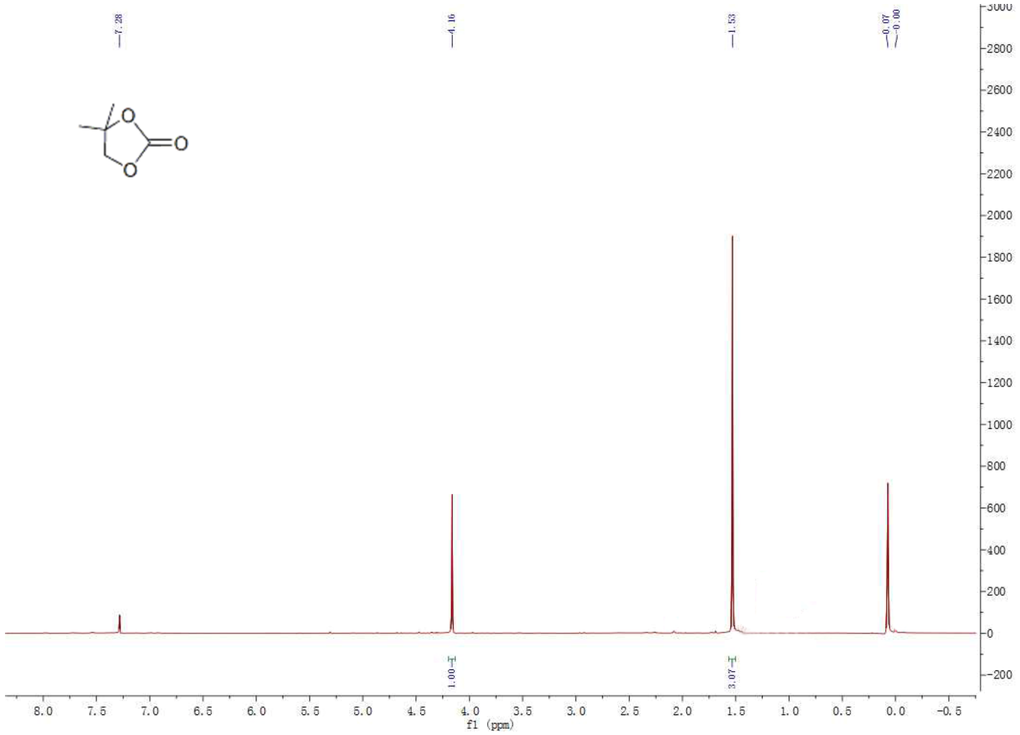

7.28
4.16
1.53
0.07
0.00
O
O
O
1.00
3.07
f1 (ppm)

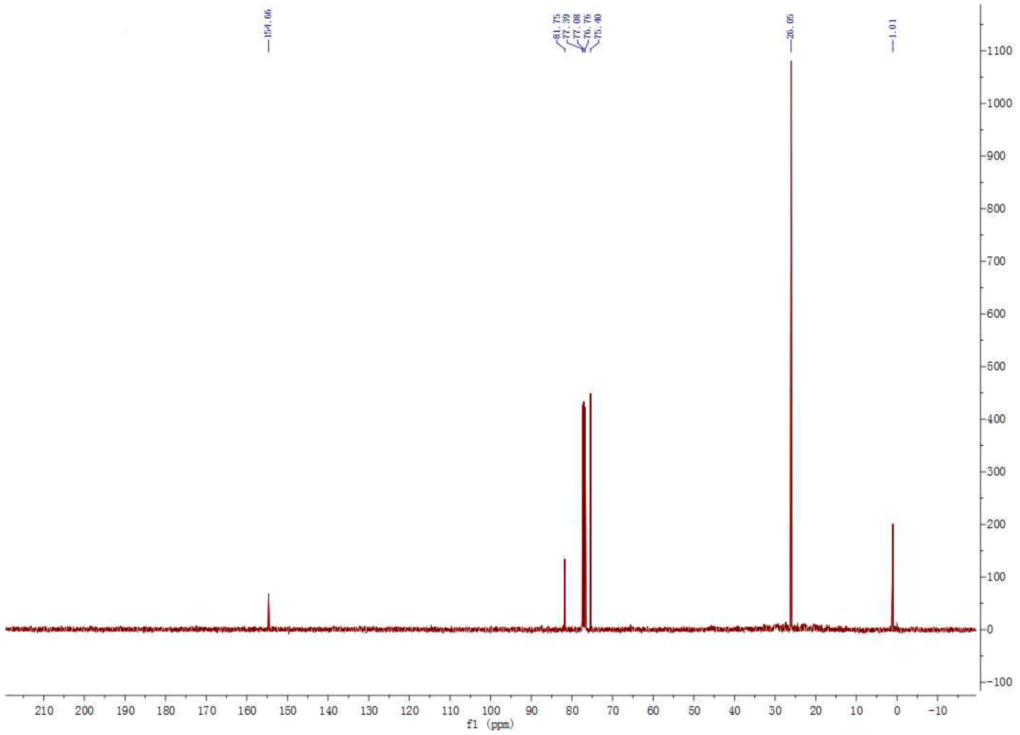

154.66
81.75
77.39
77.08
76.76
75.40
26.05
1.01
f1 (ppm)

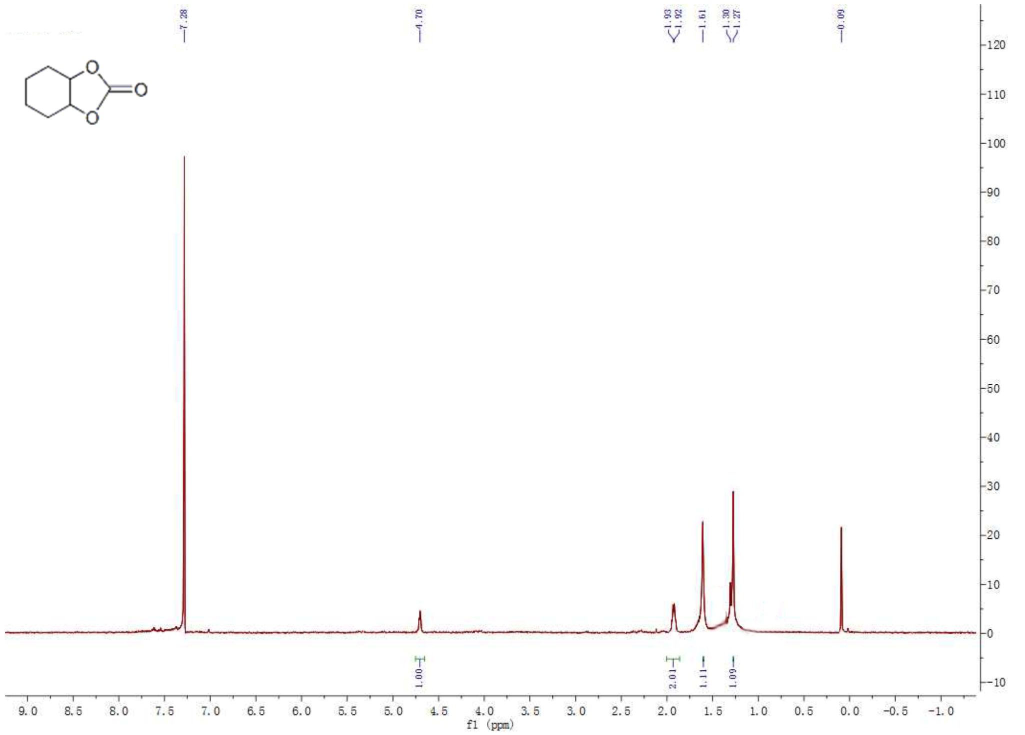

7.28
4.70
1.93
1.92
1.61
1.30
1.27
0.09
O
O
O
1.00
2.01
1.11
1.09
f1 (ppm)

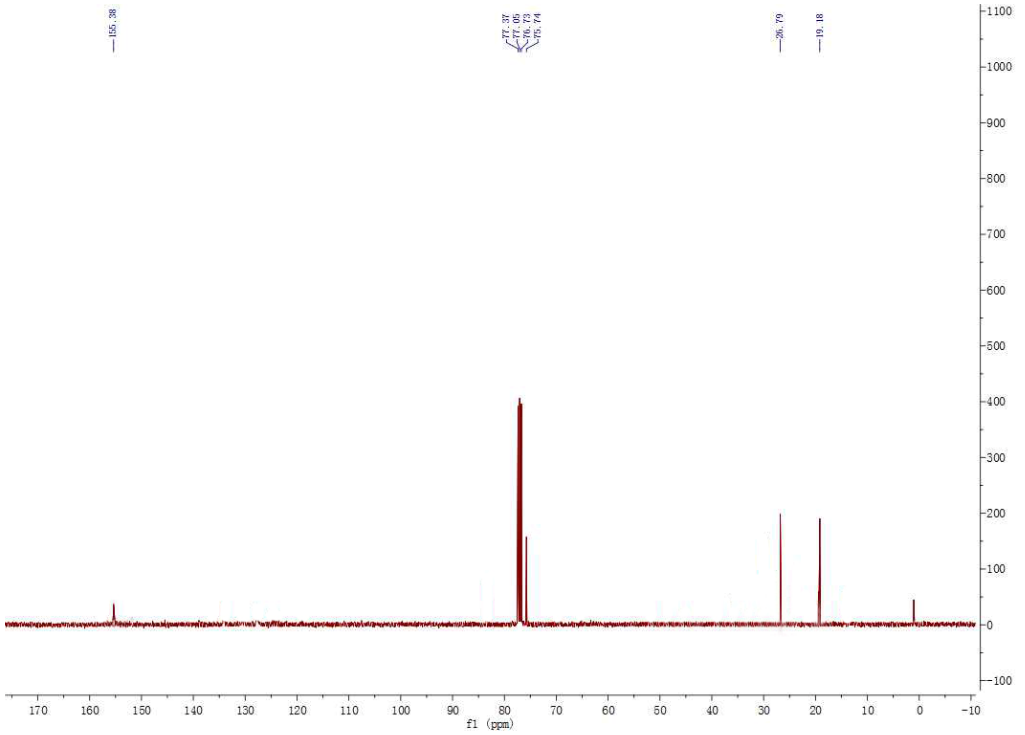

155.38
77.37
77.05
76.73
75.74
26.79
19.18
f1 (ppm)

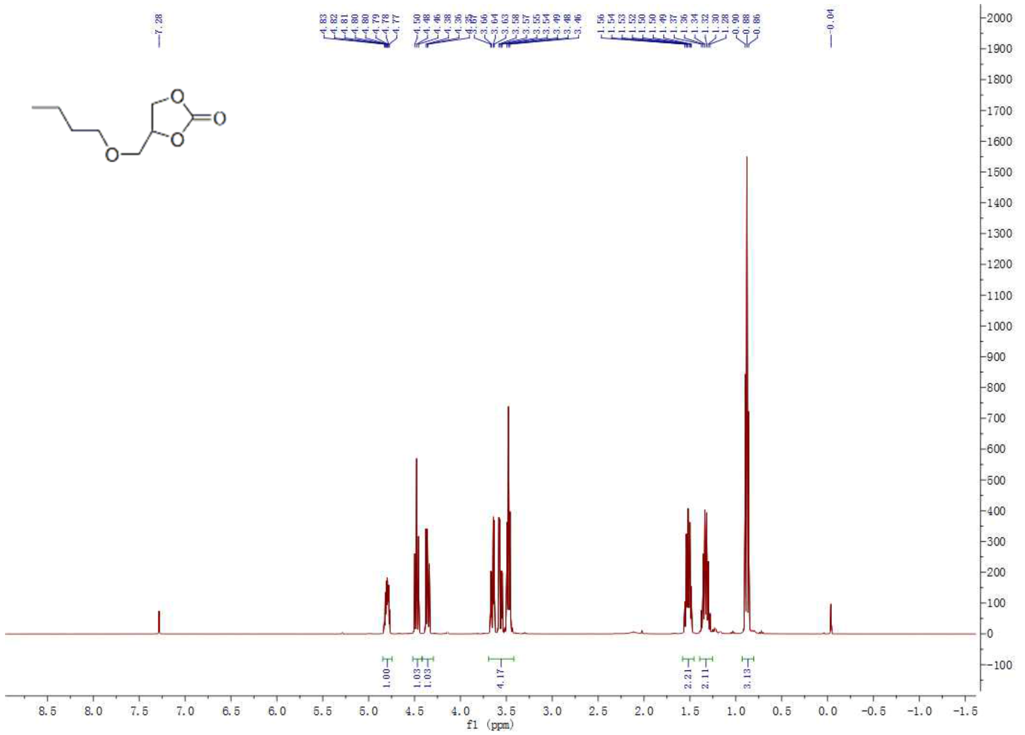

7.28
4.83
4.82
4.81
4.80
4.80
4.79
4.78
4.77
4.50
4.48
4.46
4.38
4.36
3.66
3.64
3.63
3.58
3.57
3.55
3.54
3.49
3.48
3.46
1.56
1.54
1.53
1.52
1.50
1.49
1.37
1.36
1.34
1.32
1.30
1.28
0.90
0.88
0.86
0.04
1.00
1.03
1.03
4.17
2.21
2.11
3.13
f1 (ppm)

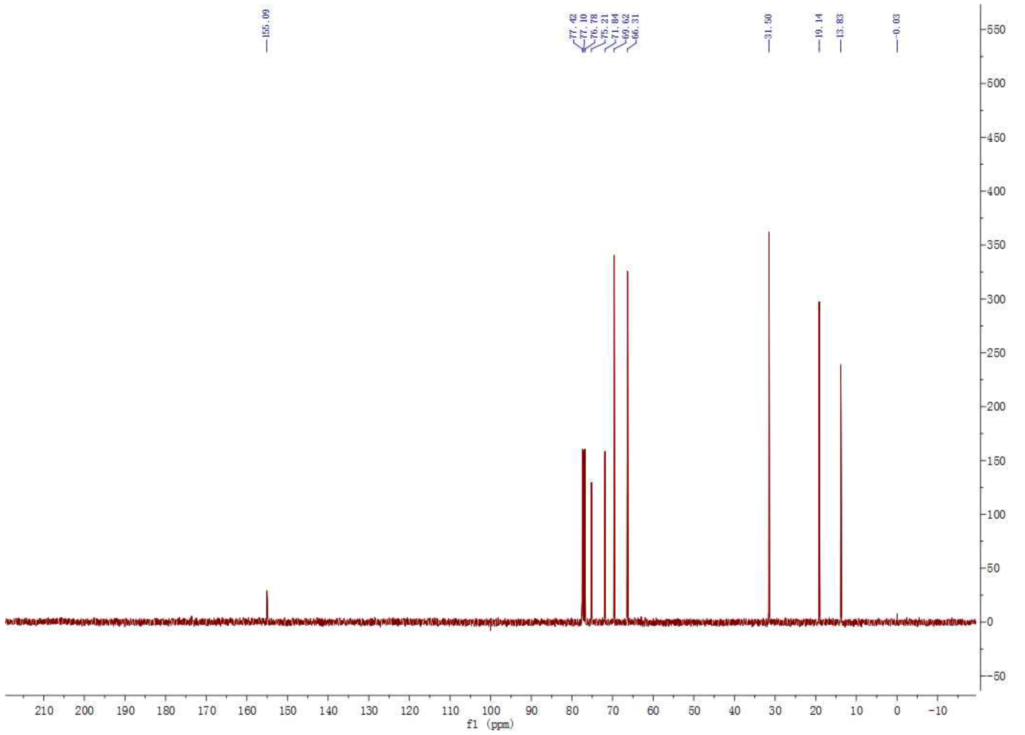

155.09
77.42
77.10
76.78
75.21
71.84
69.62
66.31
31.50
19.14
13.83
0.03
f1 (ppm)

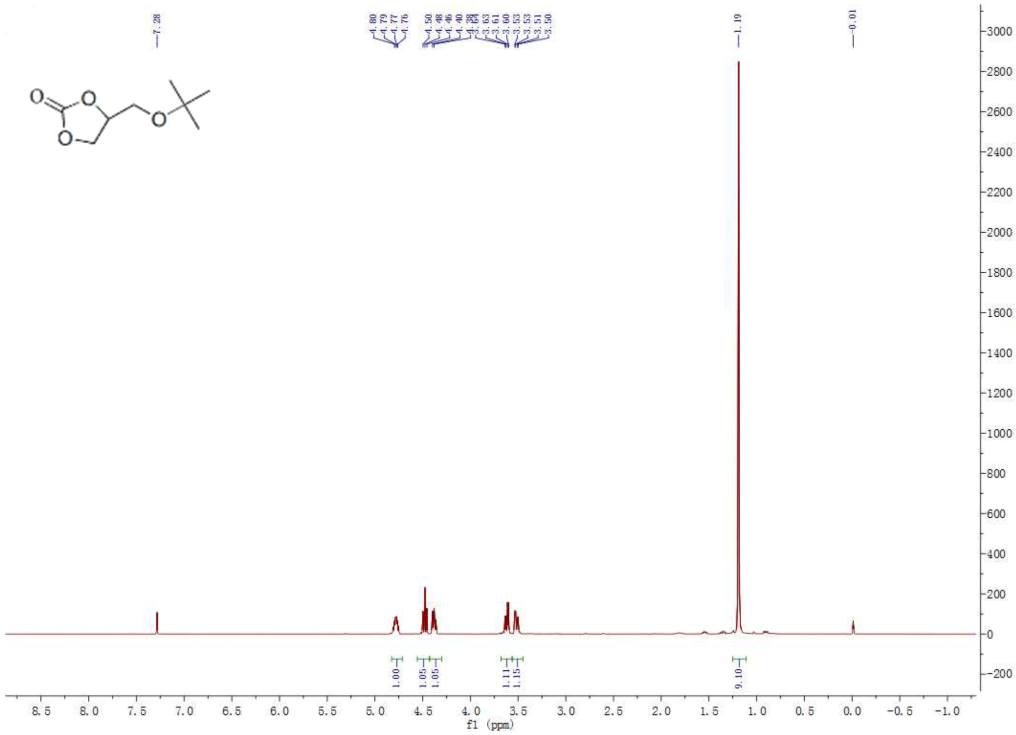
7.28
4.80
4.79
4.77
4.76
4.50
4.48
4.46
4.40
3.63
3.61
3.60
3.53
3.51
3.50
1.19
0.01
1.00
1.05
1.05
1.11
1.15
9.10
f1 (ppm)

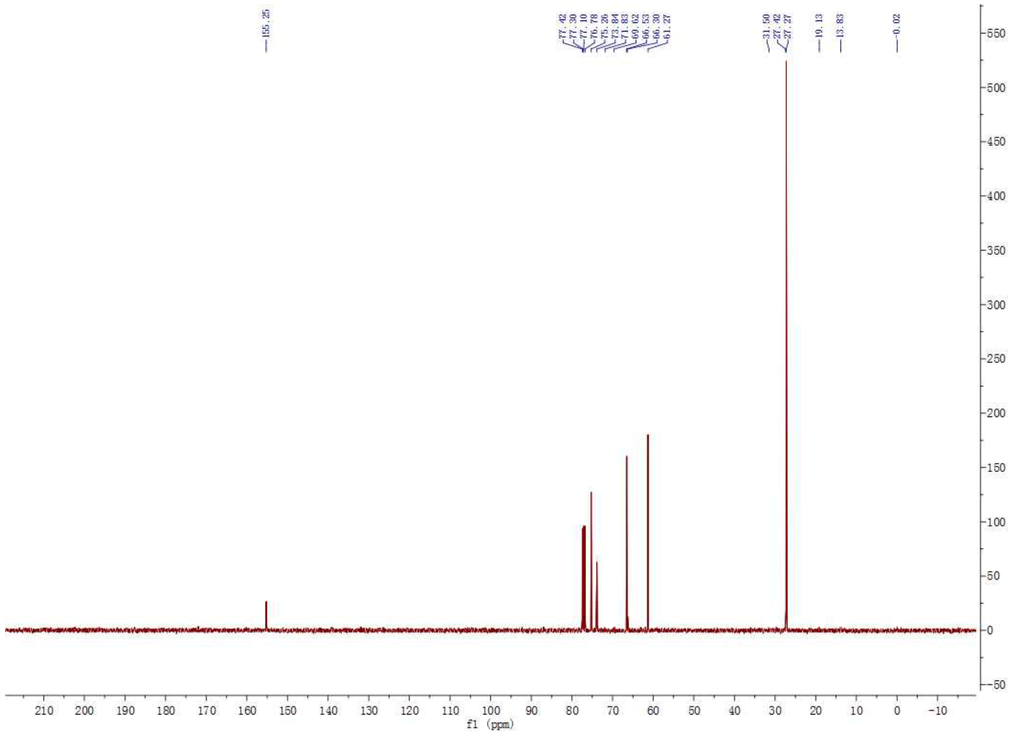
155.25
77.42
77.30
77.10
76.78
75.26
73.84
71.83
69.62
66.53
66.30
61.27
31.50
27.42
27.27
19.13
13.83
0.02
f1 (ppm)

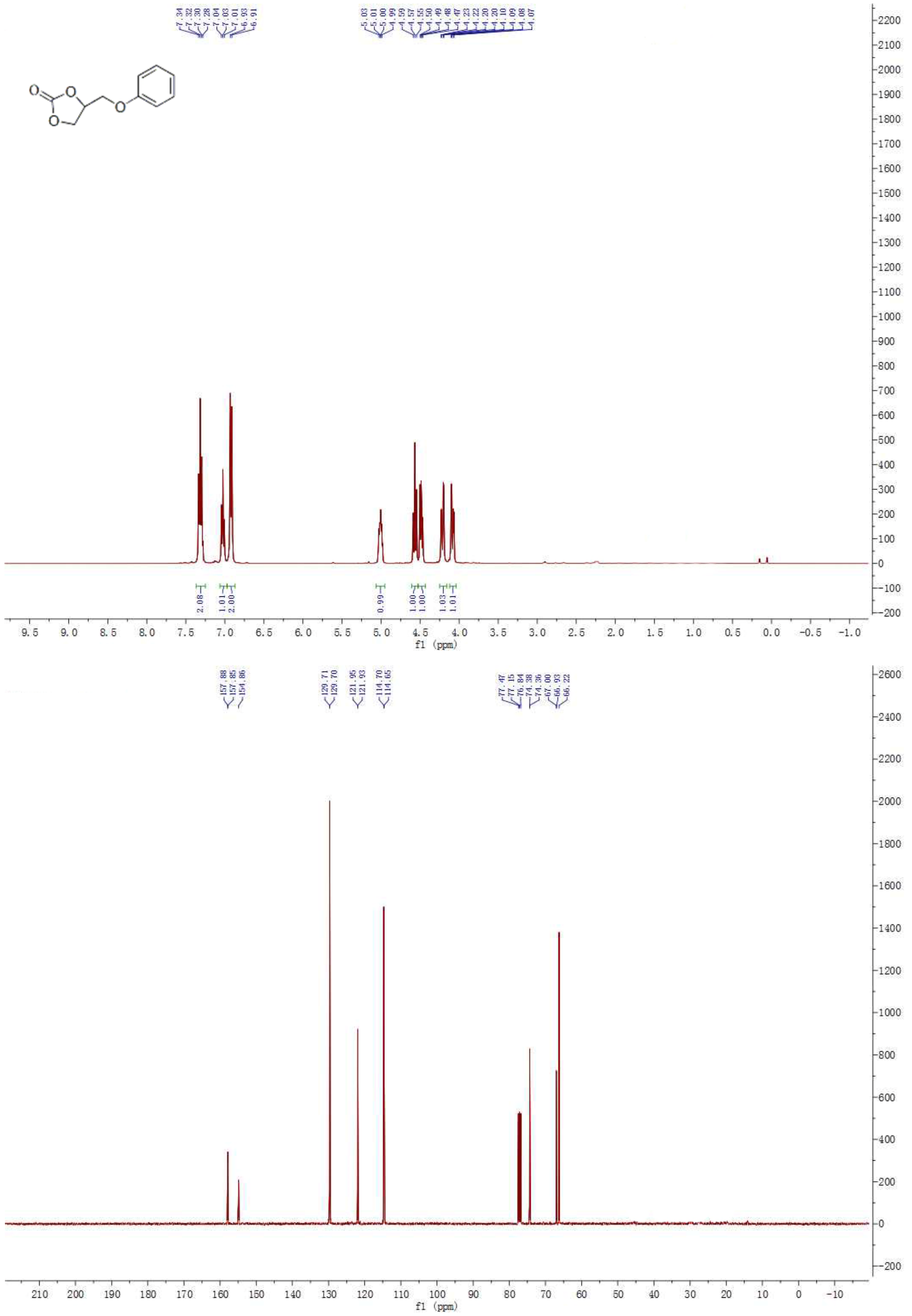

Figure S1 NMR spectra of the substances synthesized in this work.
